# Supplementary material for: Single cell regulatory landscape of the mouse kidney highlights cellular differentiation programs and disease targets
Source: Nat Commun. 2021 Apr 15;12:2277. doi: 10.1038/s41467-021-22266-1 (PMC8050063; doi:10.1038/s41467-021-22266-1)
Supplement: Supplementary file 1 — Supplementary Information [file 41467_2021_22266_MOESM1_ESM.pdf]

Supplementary Information

Single cell resolution regulatory landscape of the mouse kidney highlights cellular differentiation programs and disease targets

Zhen Miao, Michael S. Balzer, Ziyuan Ma, Hongbo Liu, Junnan Wu, Rojesh Shrestha, Tamas Aranyi, Amy Kwan, Ayano Kondo, Marco Pontoglio, Junhyong Kim, Mingyao Li, Klaus H. Kaestner & Katalin Susztak

Correspondence:
Katalin Susztak, MD, PhD
ksusztak@pennmedicine.upenn.edu

Supplementary Data

Supplementary Data 1. Quality control and metadata information of scRNA-seq data.
Supplementary Data 2. Cell type marker genes derived from scRNA-seq analysis & marker genes used for annotation.
Supplementary Data 3. DEG in scRNA-seq data after ambient RNA cleaning.
Supplementary Data 4. DEG in scRNA-seq data of stroma subclusters.
Supplementary Data 5. Cell type-specific open chromatin derived from snATAC-seq analysis.
Supplementary Data 6. Cell type-specific motif enrichment.
Supplementary Data 7. Regulons and respective target genes inferred by SCENIC.
Supplementary Data 8. Scaled and binarized regulon activities in each cell type inferred by SCENIC.
Supplementary Data 9. ChromVAR cell-TF enrichment score matrix.
Supplementary Data 10. DEG along pseudotime in distinct lineages in scRNA-seq data.
Supplementary Data 11. Differentially accessible peaks along pseudotime in distinct lineages in snATAC-seq data.
Supplementary Data 12. Nearest genes of differentially accessible peaks along pseudotime in distinct lineages in snATAC-seq data.
Supplementary Data 13. GO enrichment of differentially accessible peaks along pseudotime in distinct lineages inferred by GREAT analysis.
Supplementary Data 14. Nearest genes of differentially accessible peaks at bifurcation events along pseudotime in distinct lineages inferred by GREAT analysis.
Supplementary Data 15. Proportion of cells in each cell type with accessible chromatin overlapped with kidney disease-associated SNPs.
Supplementary Data 16. Material Table

Supplementary Figures

Pages 2-26

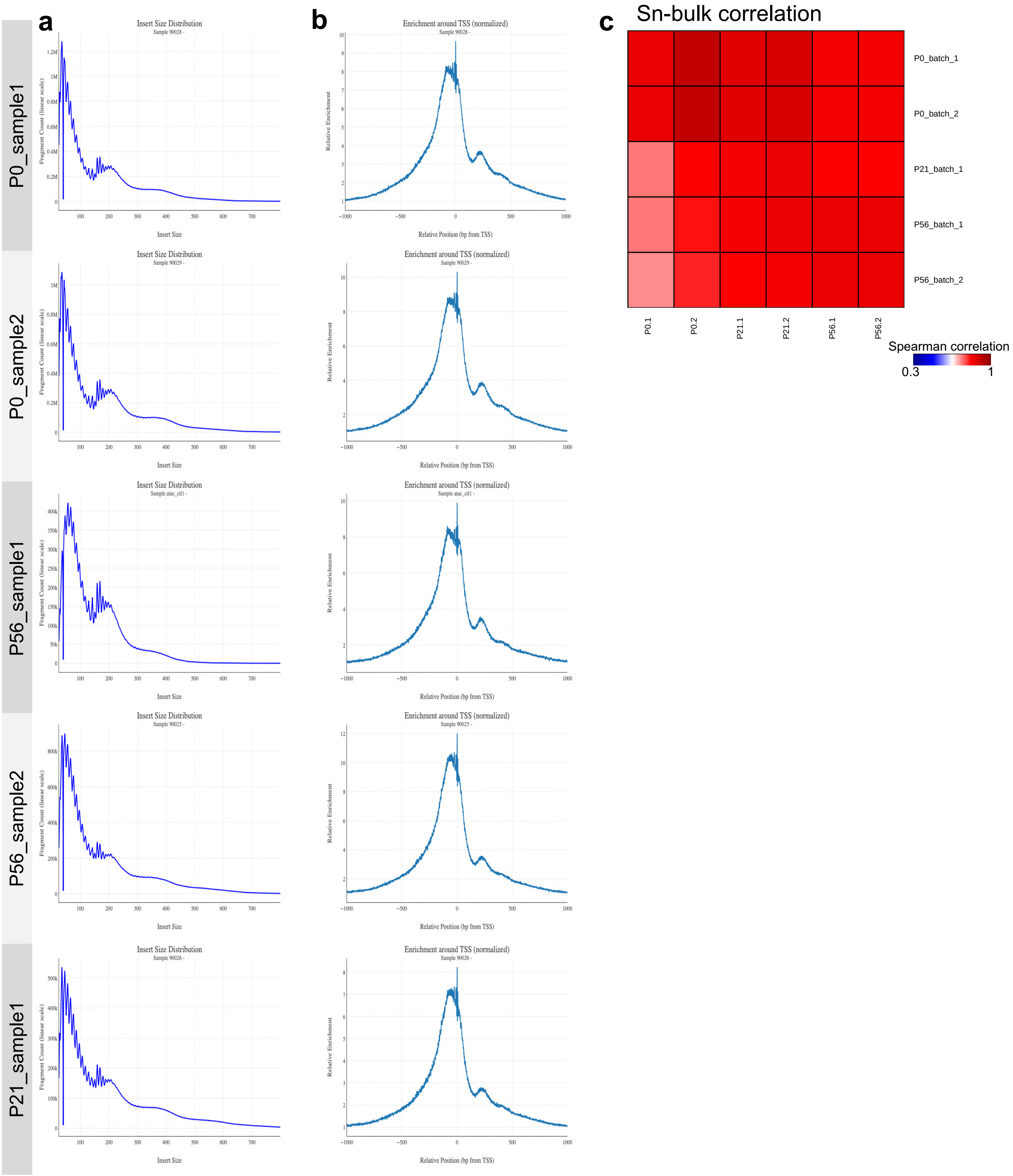

Supplementary Figure 1. Quality control for snATAC-seq data.

(a) Insert size distribution of the 5 snATAC-seq samples showing periodic patterns. (b) Transcription start sites (TSS) signal enrichment of the 5 snATAC-seq samples. (c) Spearman correlation between snATAC-seq datasets and bulk ATAC-seq of binned genomic regions.

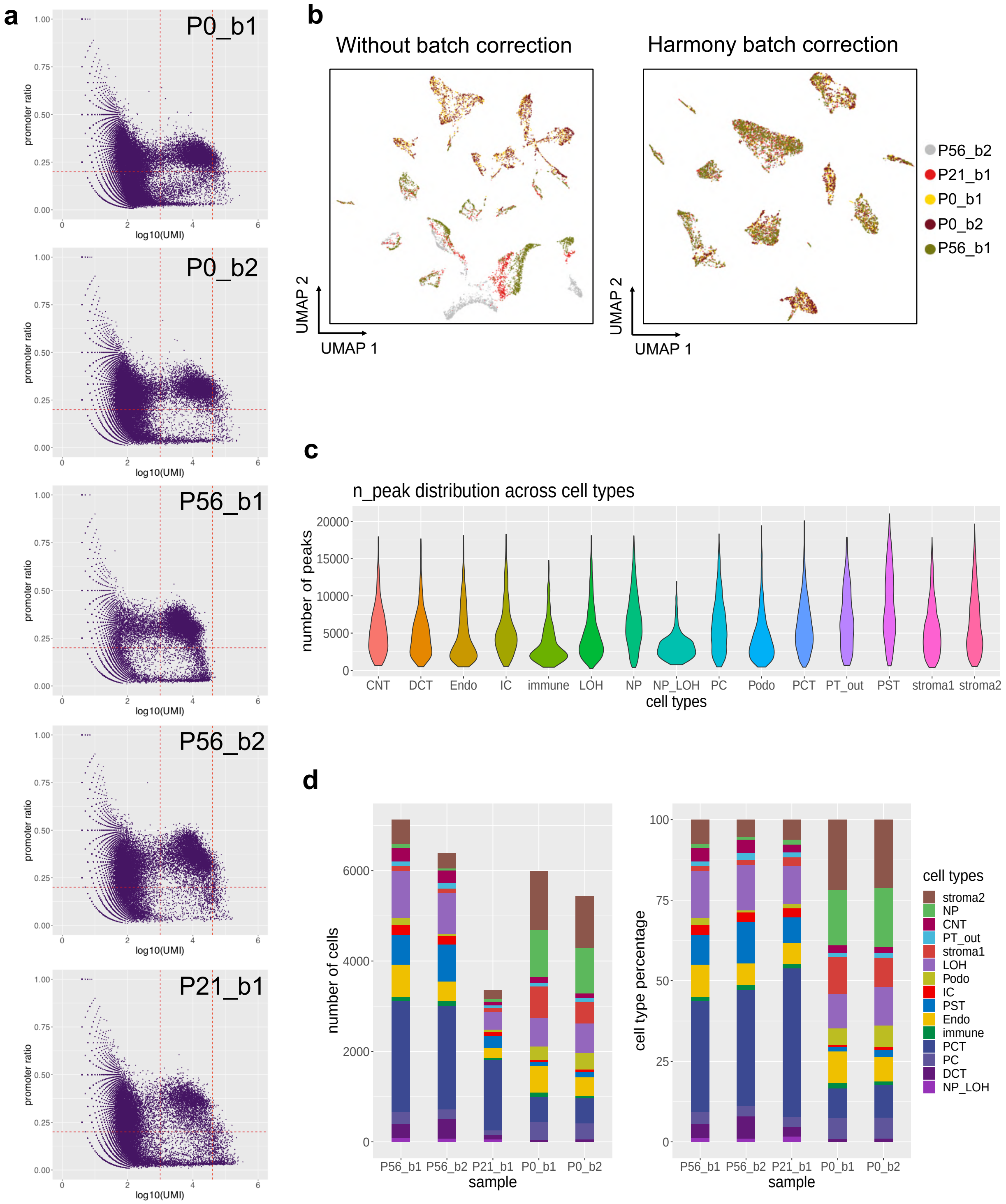

Supplementary Figure 2. Quality control and data processing for snATAC-seq data.
(a) Distribution of number of unique molecular identifiers (UMIs, x axis) and promoter ratio (y axis) in 5 samples shown by dot plot. (b) UMAP representation of the snATAC-seq dataset before and after batch correction. (c) Violin plots representing the number of accessible peaks across different clusters in the snATAC-seq dataset indicating similar distributions. (d) Stacked bar graphs representing absolute numbers and percentages of identified cell types across snATAC-seq batches.

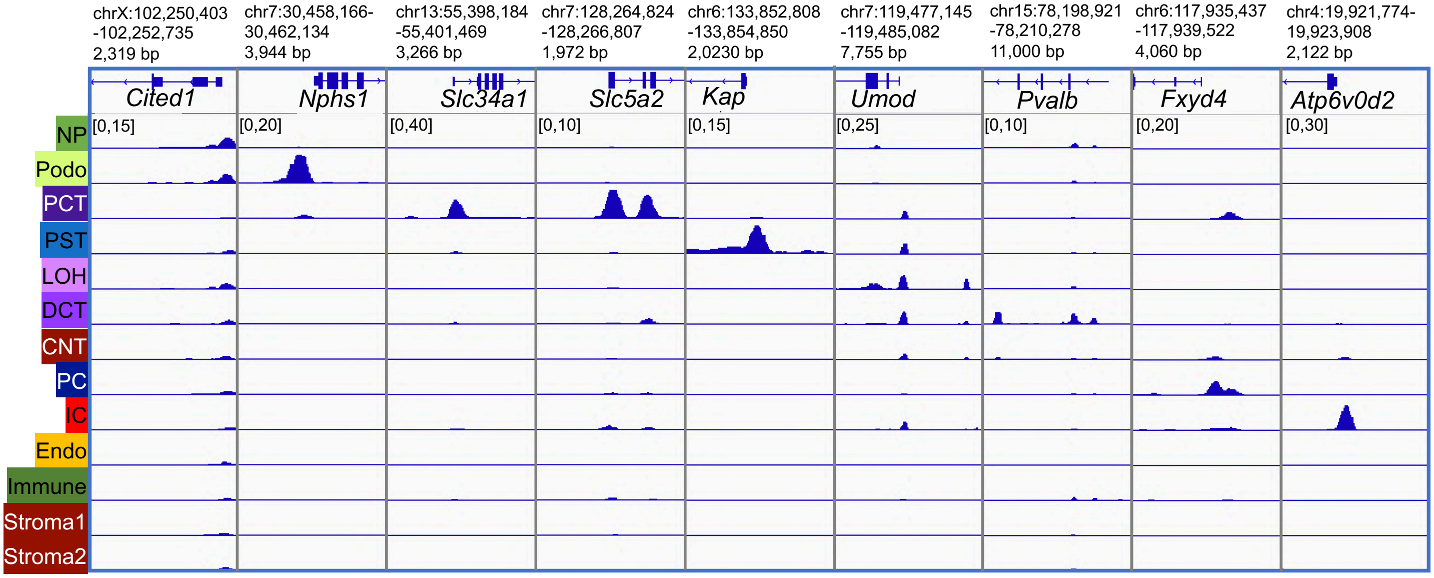

Supplementary Figure 3. Cell type-specific snATAC-seq peaks.

Genome browser view of cell type-specific peaks at the TSS of marker genes for 13 cell types in the snATAC-seq dataset.

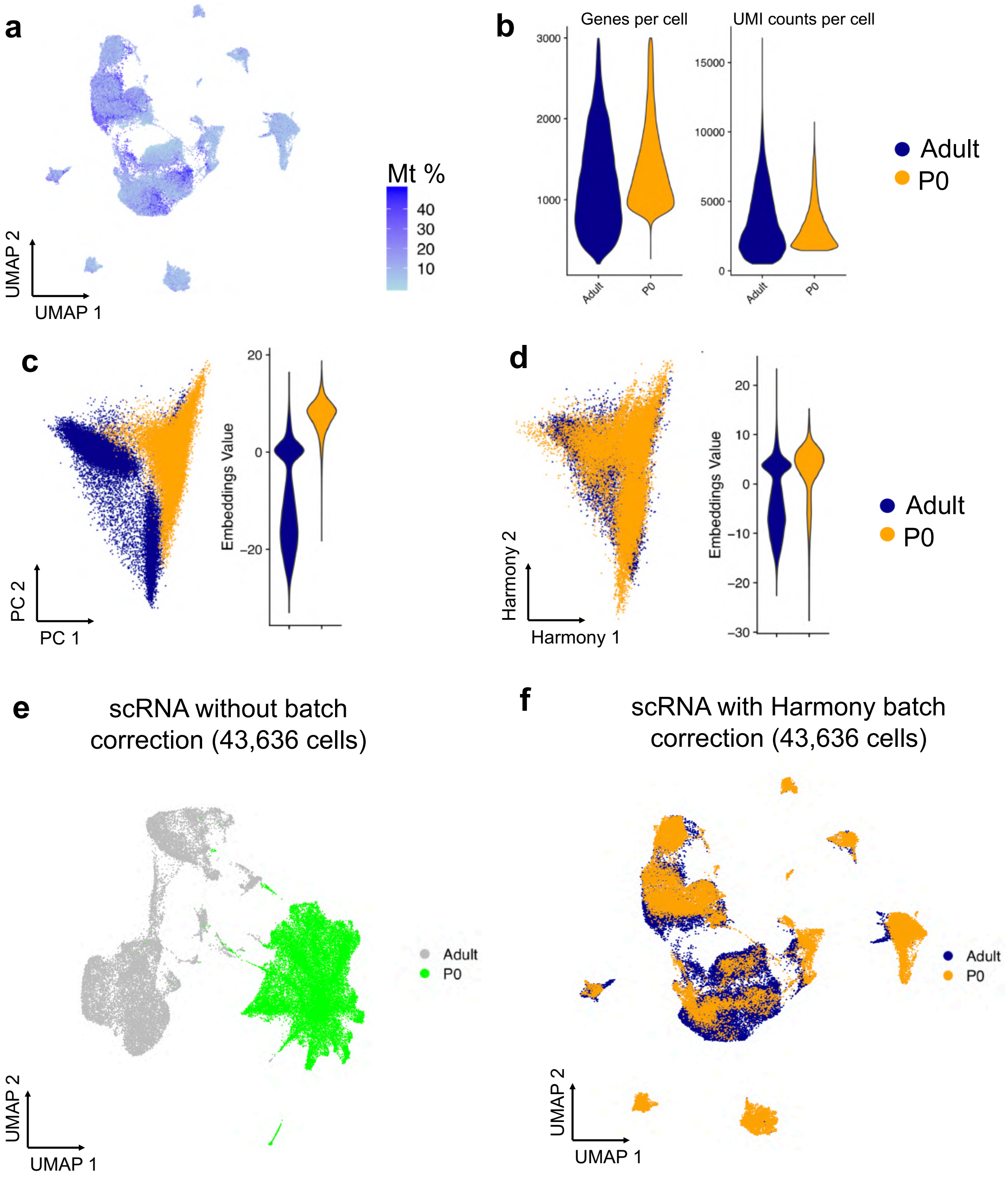

Supplementary Figure 4. Quality control and batch correction of scRNA-seq data.
(a) UMAP representation of scRNA-seq data colored by the mitochondrial gene ratio. (b) Violin plots showing number of informative genes per single cell and unique molecular identifiers (UMIs) per single cell. Blue denotes adult kidney, orange denotes P0 kidney. (c, d) Principal component (PC) representation of combined adult and P0 scRNA-seq dataset (left panel) and violin plots of corresponding embeddings values (right panel) before and after batch correction using Harmony. (e) UMAP plot visualizing 43,636 cells without batch correction. Grey denotes adult, green denotes P0 cells. (f) UMAP plot visualizing 43,636 cells following Harmony batch correction. Blue denotes adult, orange denotes P0 cells.

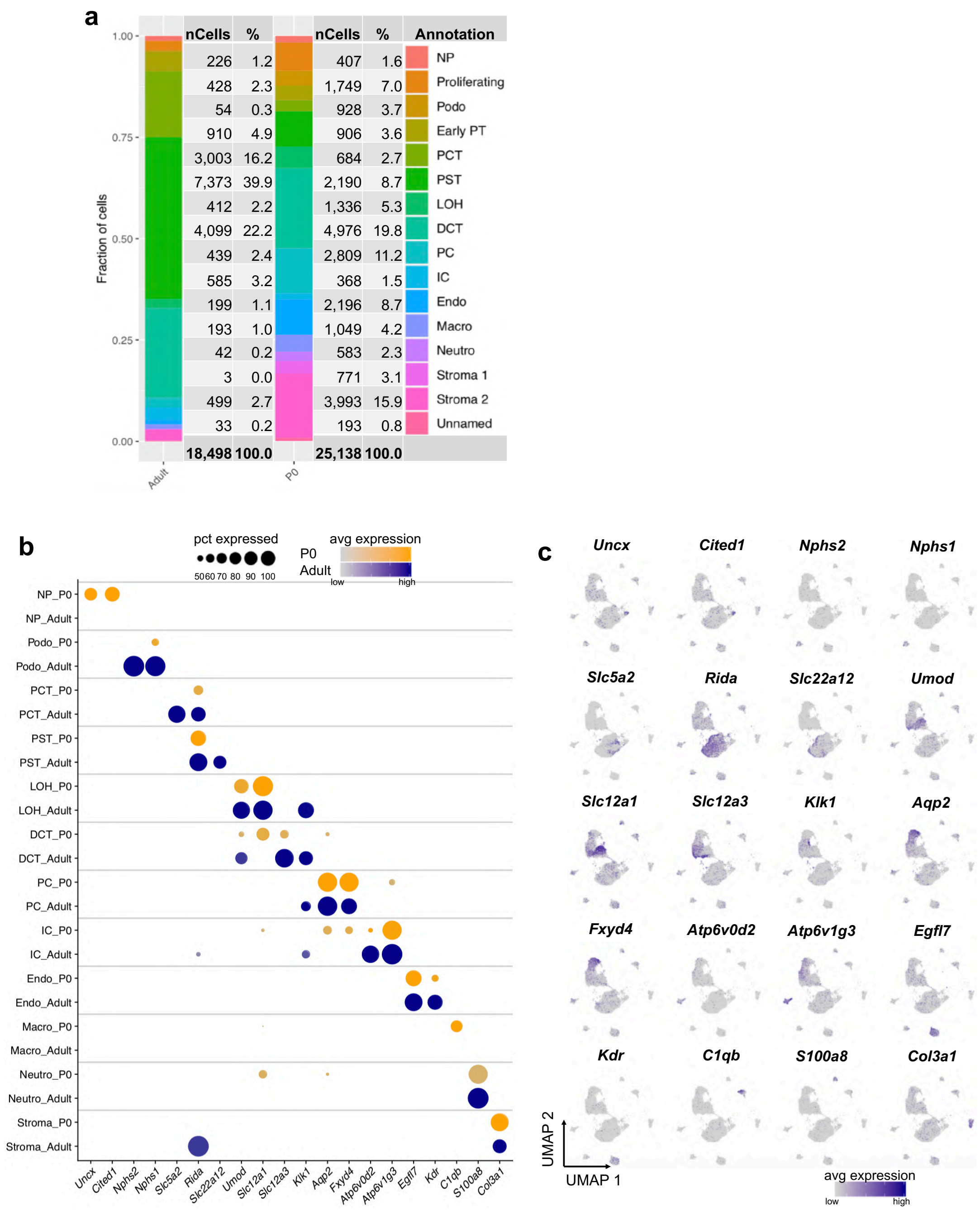

Supplementary Figure 5: P0 and adult scRNA-seq cell-type annotation.

(a) From left to right: Stacked bar graphs showing the percentage of different cell types in the adult and P0 scRNA-seq datasets, tables showing the number of cells in each cell type (nCells) and corresponding percentage. NP, nephron progenitor; Podo, podocyte; PCT, proximal convoluted tubule; PST, proximal straight tubule; LOH, loop of Henle; DCT, distal convoluted tubule cells; PC, collecting duct principal cells; IC, collecting duct intercalated cells; Endo, endothelial cells; Macro, macrophages; Neutro, neutrophils. (b) Dot plot of cell type-specific marker genes. Dot size denotes percentage of cells expressing the marker. Color scale represents average expression, orange denotes P0, blue denotes adult kidney. (c) Feature plots of representative marker genes projected on UMAP dimension.

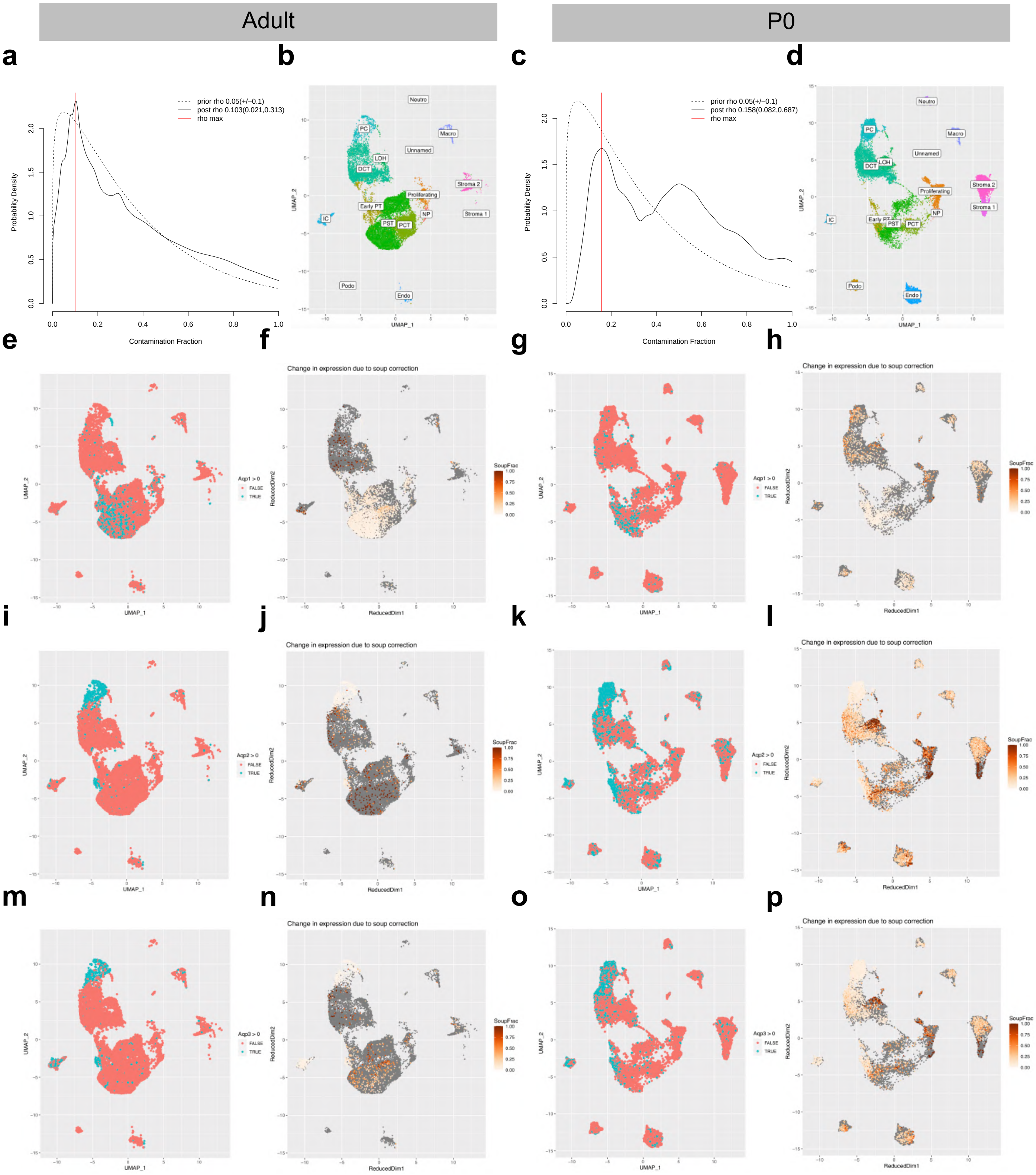

Adult
P0
a
b
c
d
e
f
g
h
i
j
k
l
m
n
o
p
prior rho 0.05(+/−0.1)
post rho 0.103(0.021,0.313)
rho max
post rho 0.158(0.082,0.687)
Probability Density
Contamination Fraction
UMAP_1
UMAP_2
Neutro
Macro
Unnamed
PC
LOH
DCT
Stroma 2
Proliferating
Stroma 1
Early PT
NP
PST
PCT
IC
Podo
Endo
Change in expression due to soup correction
Aqp1 > 0
Aqp2 > 0
Aqp3 > 0
FALSE
TRUE
SoupFrac
ReducedDim1
ReducedDim2

Supplementary Figure 6: Ambient RNA correction in scRNA-seq data.
(a, c) Estimated fractions of ambient RNA contamination in adult (a) and P0 (c) batches, respectively. Cells contributing to the combined UMAP representation are visualized separately for adult (b) and P0 batches (d). (e, i, m, g, k, o) Cells from adult and P0 samples demonstrating non-zero expression of *Aqp1*, *Aqp2* and *Aqp3* before correction for ambient RNA are visualized in UMAP space. Red denotes negative, green denotes positive cells. (f, j, n, h, l, p) Corresponding UMAP plots showing the change of expression due to correction for ambient RNA (soup). Cells with a high fraction of ambient RNA are colored red, cells with low ambient RNA fraction are colored white.

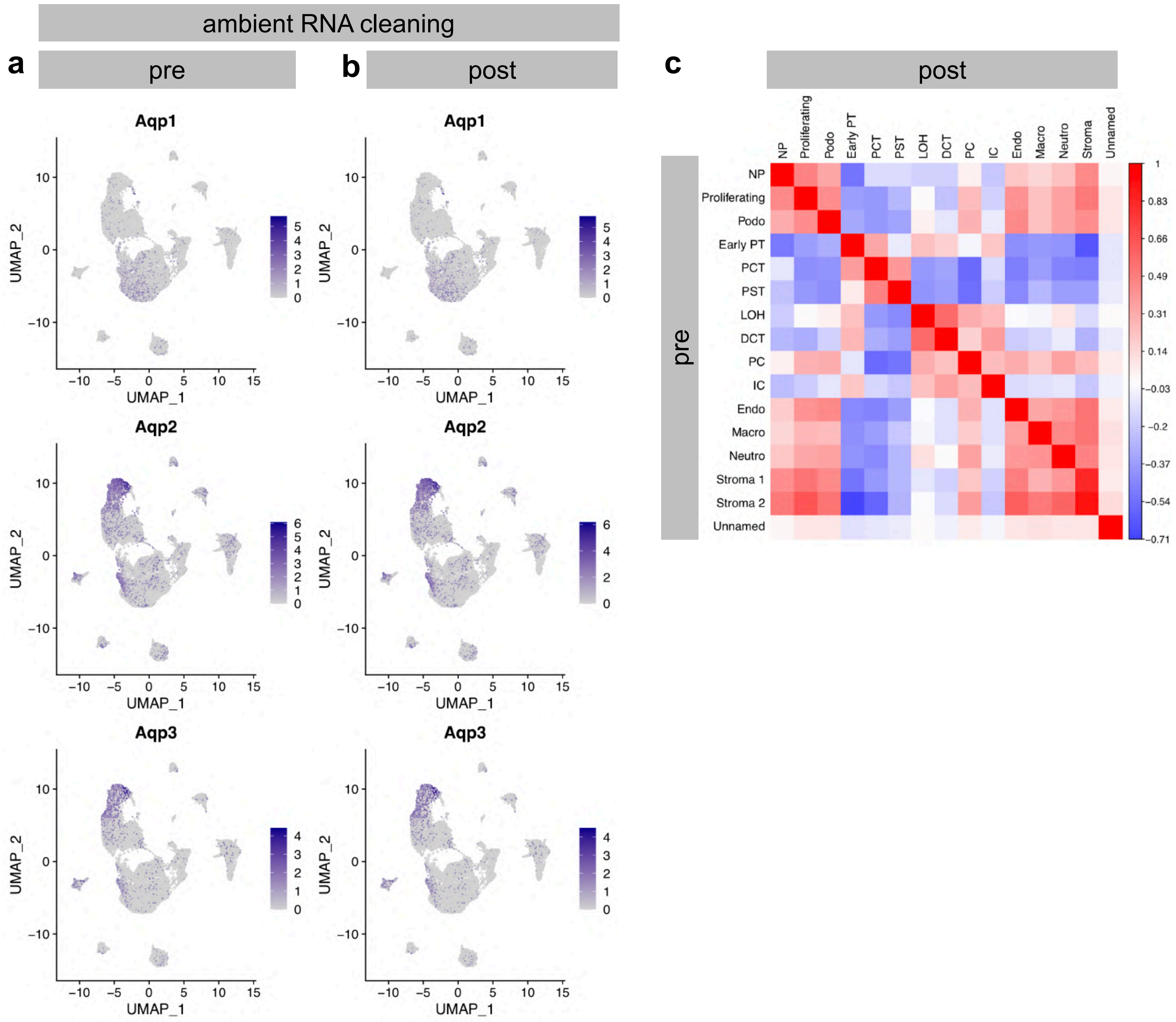

Supplementary Figure 7: Comparison of scRNA-seq data processing with and without ambient RNA cleaning.

(a, b) UMAP plots visualizing *Aqp1*, *Aqp2* and *Aqp3* in the combined dataset (P0 and adult) with (b) and without (a) cleaning for ambient RNA. Note: For comparison purposes, expression after correction for ambient RNA (b) is projected onto UMAP coordinates resulting from dimension reduction without ambient RNA correction. (c) Correlation matrix visualizing Pearson correlation coefficients (PCC) between average cell type gene expressions of the original matrix without correction for ambient RNA and the matrix corrected for ambient RNA.

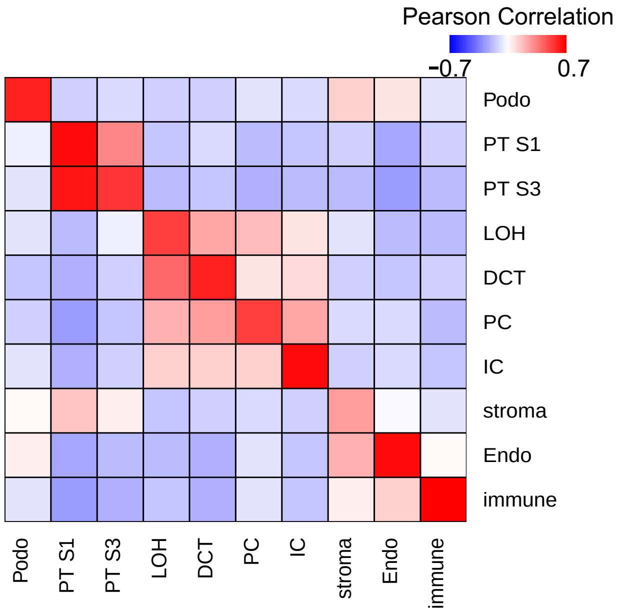

Supplementary Figure 8: Correlation between gene expression and chromatin accessibility
Heatmap showing Pearson's correlation coefficients between snATAC-seq gene activity scores and gene expression values in adult data, which is complementary to **Figure 1g** depicting P0 data. Each row represents a cell type in scRNA-seq data and each column represents a cell type in snATAC-seq data.

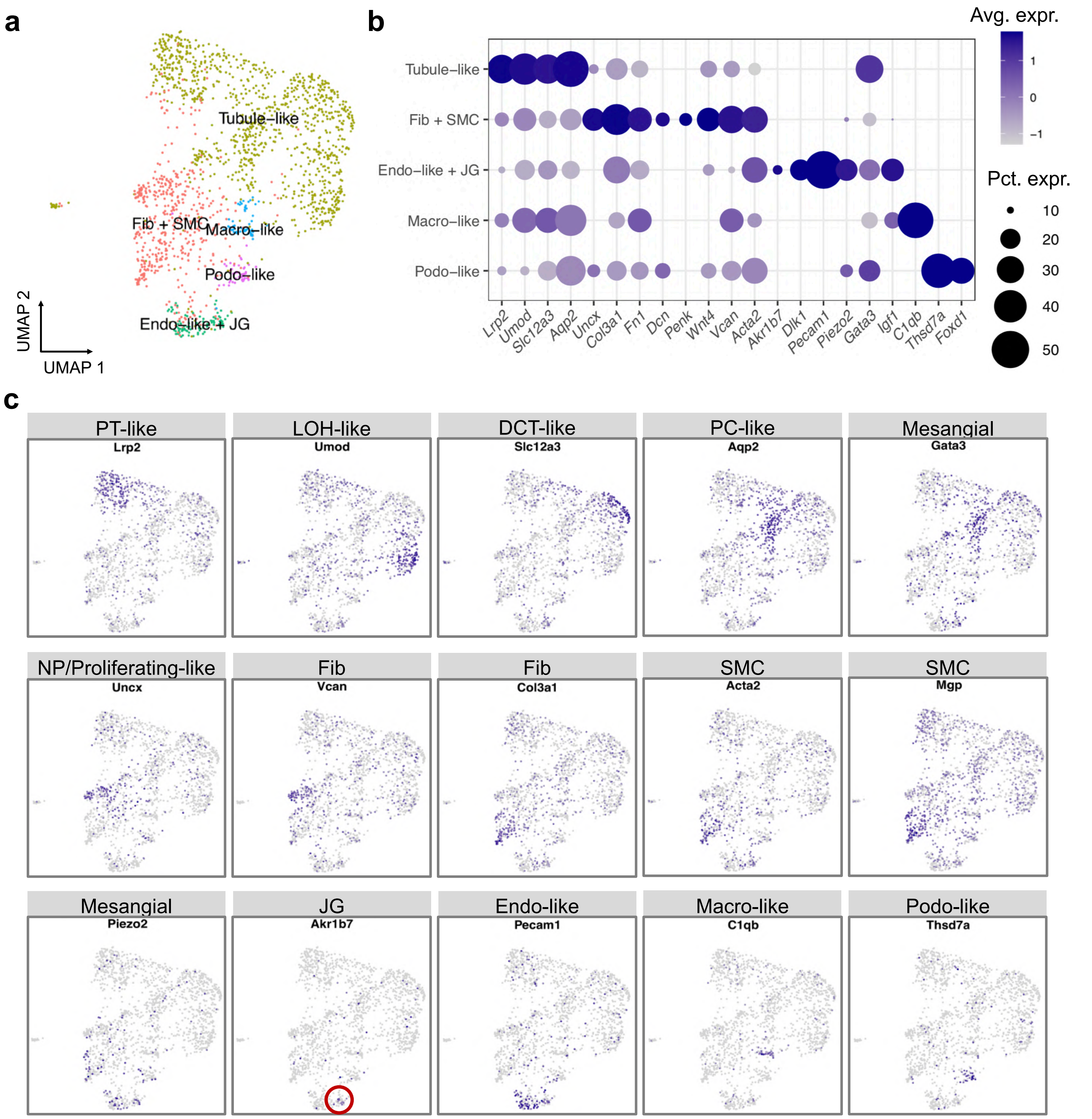

Supplementary Figure 9: P0 stroma cell subclustering.

(a) UMAP plot visualizing subclustering P0 mouse kidney of stromal cells. SMC, smooth muscle cell; Fib, fibroblast; Podo, podocyte; Endo, endothelial cell; Macro, macrophage. (b) Dotplot showing gene expression levels in the stroma subclusters. Note that a small fraction of cells in "Endo + JG" cluster demonstrates juxtaglomerular (JG) marker gene expression. (c) Corresponding featureplots for markers in (b).

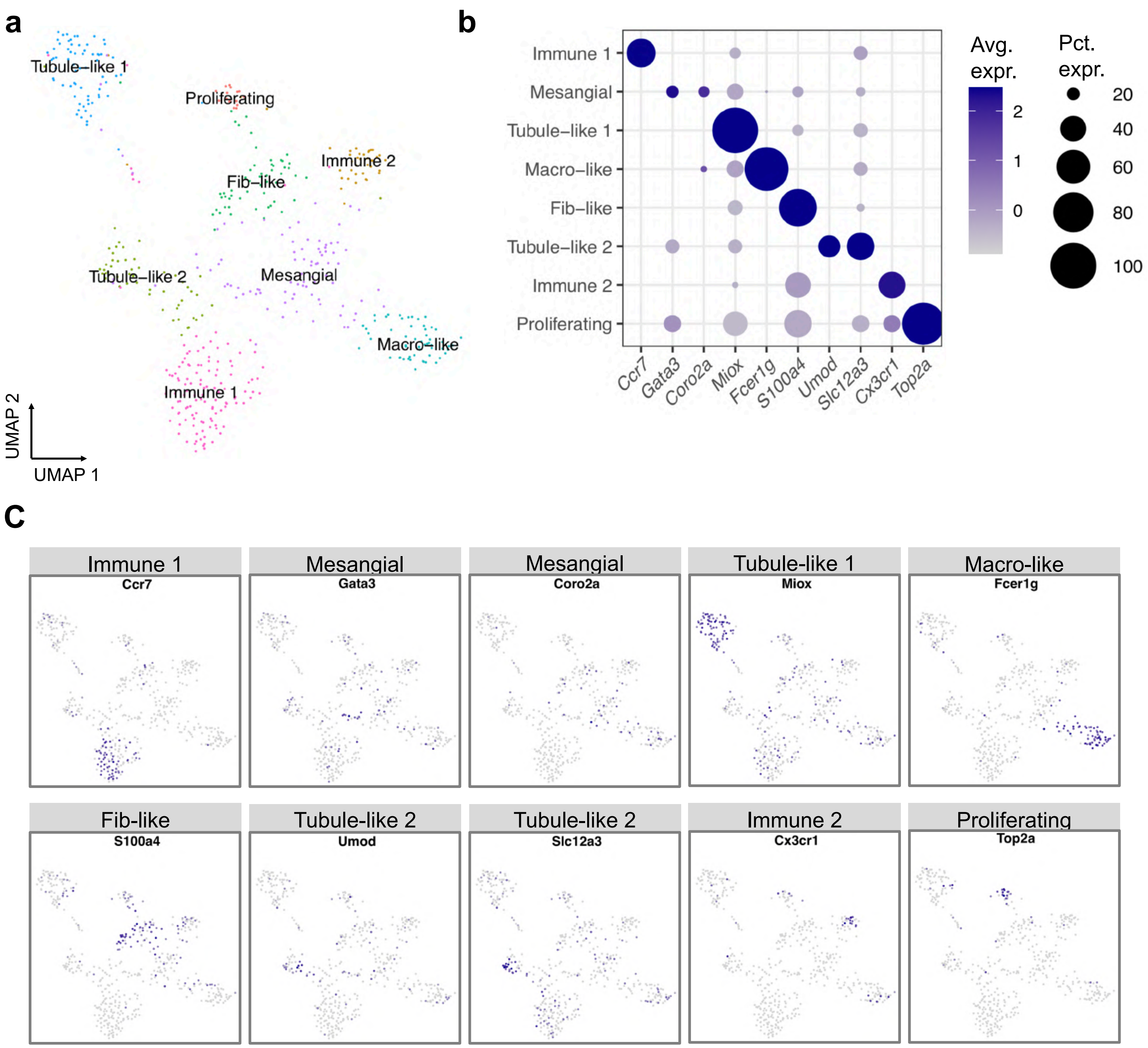

Supplementary Figure 10: Adult stroma cell subclustering.
(a) UMAP plot visualizing subclustering of adult mouse kidney stromal cells. (b, c) Corresponding dotplot and featureplots demonstrate considerable overlap of marker gene expression.

Interactive website: susztaklab.com/developing_adult_kidney/igv/

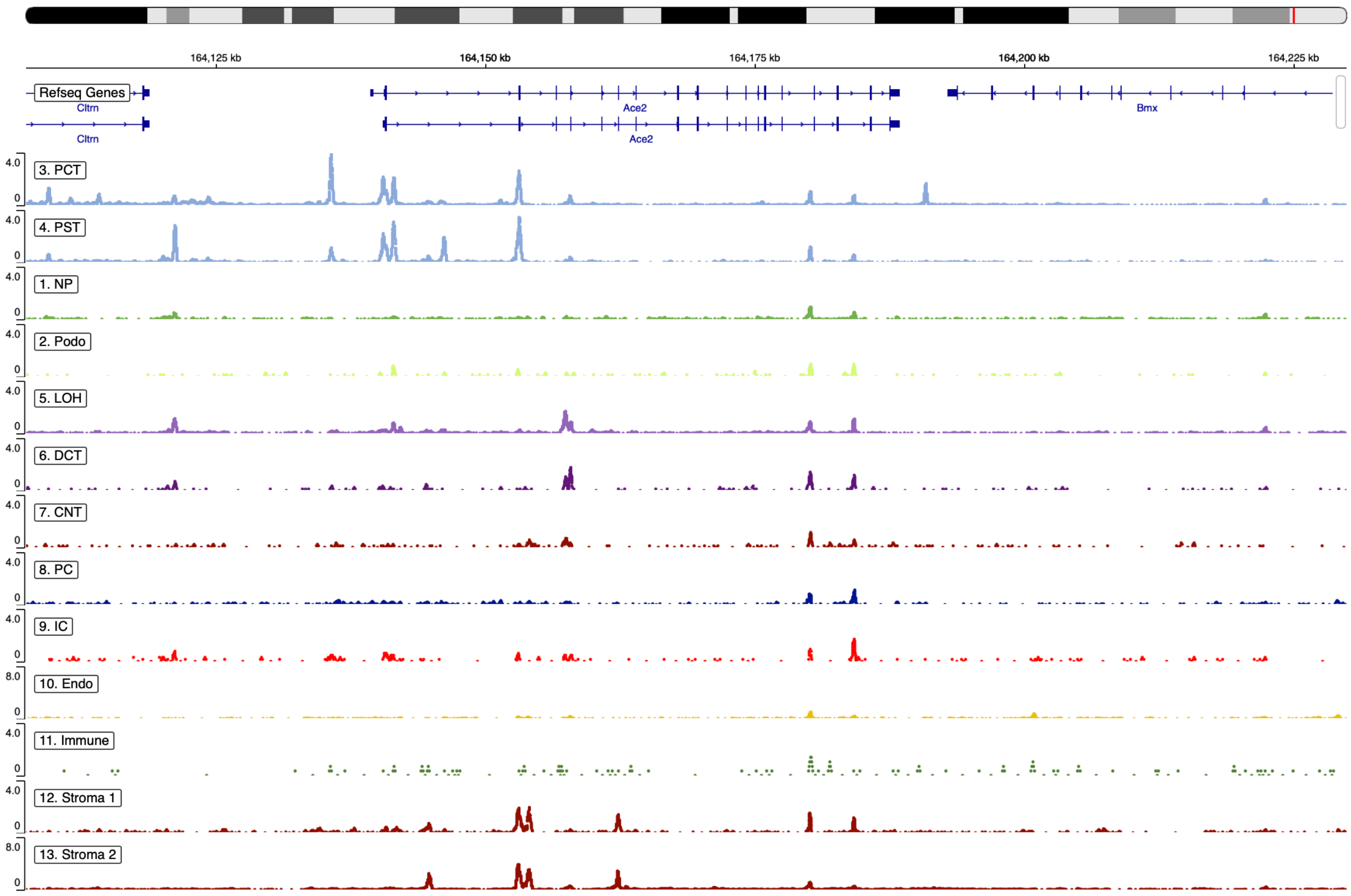

Supplementary Figure 11: Interactive website for chromatin accessibility.
We provide the processed chromatin accessibility dataset via a searchable, interactive website (susztaklab.com/developing_adult_kidney/igv/). In this example, we show proximal tubule-specific enrichment of accessible peaks at TSS of *Ace2* gene.

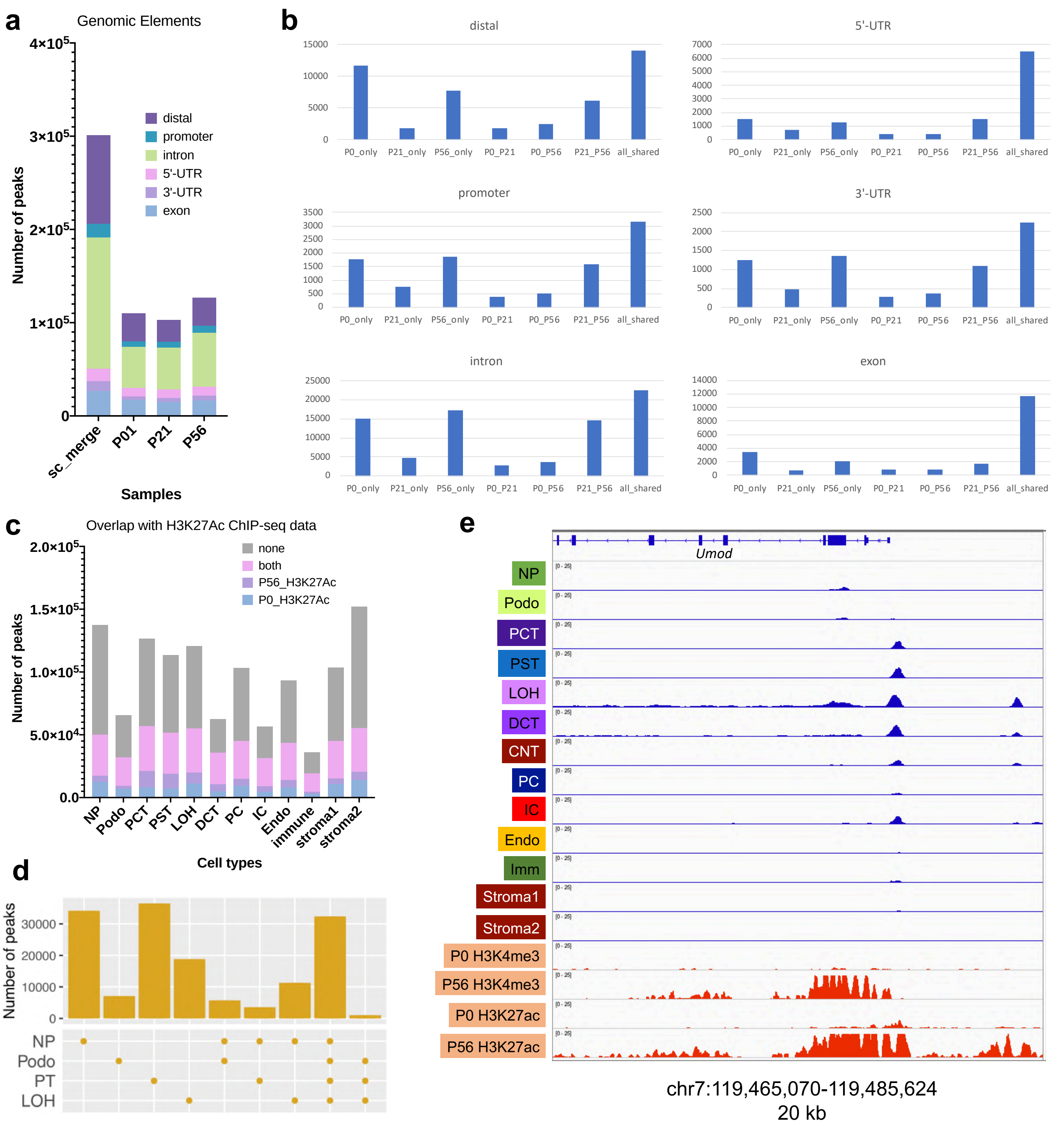

Supplementary Figure 12. Characterization of the cell type-specific regulatory landscape.
(a, b) Bar graphs representing the number of accessible peaks in distal elements, promoters, introns, 5'-UTR, 3'UTR and exons, as distributed across samples of snATAC-seq data and bulk ATAC-seq data. (c) Overlap of scATAC-seq differentially accessible peaks among cell types with H3K27Ac ChIP-seq data. (d) Number of shared and unique peaks among snATAC-seq cell types. Cell types include nephron progenitors and cells differentiated from nephron progenitors. (e) Genome browser view of *Umod* as an example for distal open chromatin region and its target promoter region.

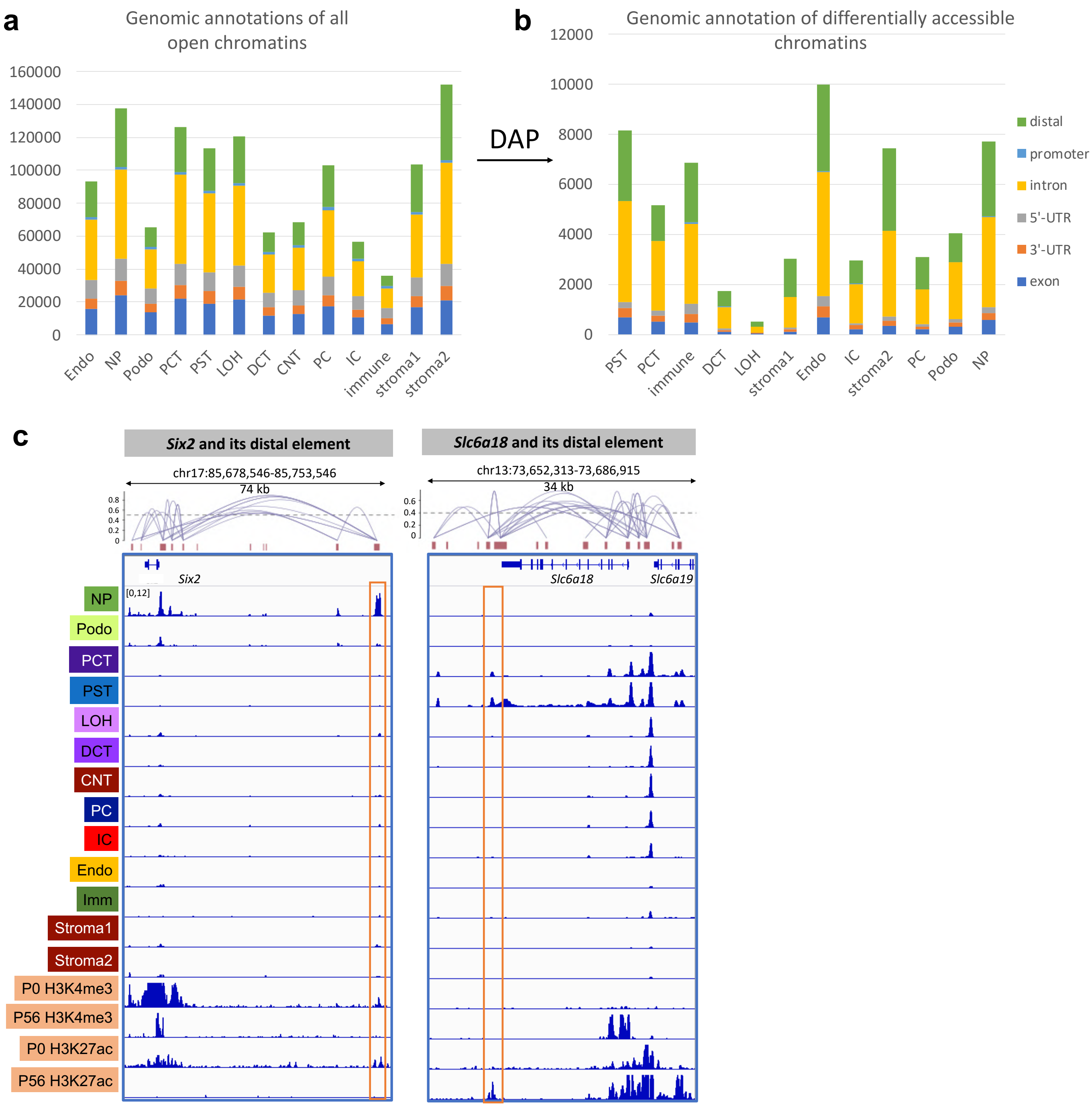

Supplementary Figure 13. Genomic annotations of open chromatins.

(a) Distribution of open chromatin elements in snATAC-seq cell types. (b) Distribution of open chromatin elements among differentially accessible peaks (DAPs) in snATAC-seq cell types. (c) Genome browser representations of single nuclei open chromatin data for individual cell types at chromosomal loci around *Six2* and *Slc6a18*, along with their known distal elements (red boxes). Corresponding chromosomal interaction of open chromatin regions, as inferred by Cicero (**Methods**), is depicted at the top along with the respective genomic region.

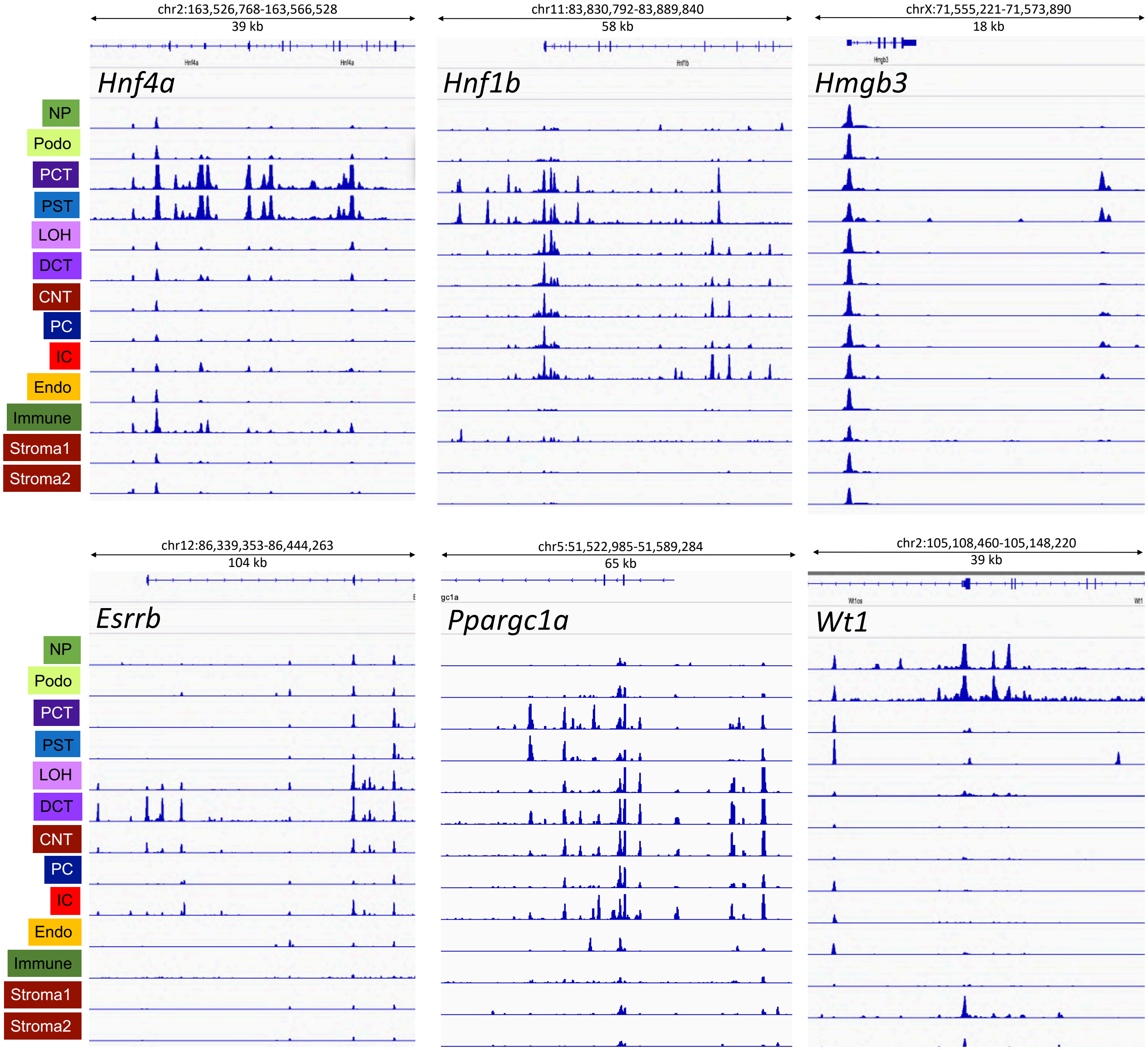

Supplementary Figure 14. Examples of cell type-specific chromatin accessibility.
Genome browser views of representative marker genes demonstrating cell type-specific chromatin accessibility for proximal tubule (*Hnf4a* and *Hmgb3*), several tubular segments (*Hnf1b*), loop of Henle and distal convoluted tubule (*Esrrb* and *Ppargc1a*) as well as nephron progenitors and podocytes (*Wt1*).

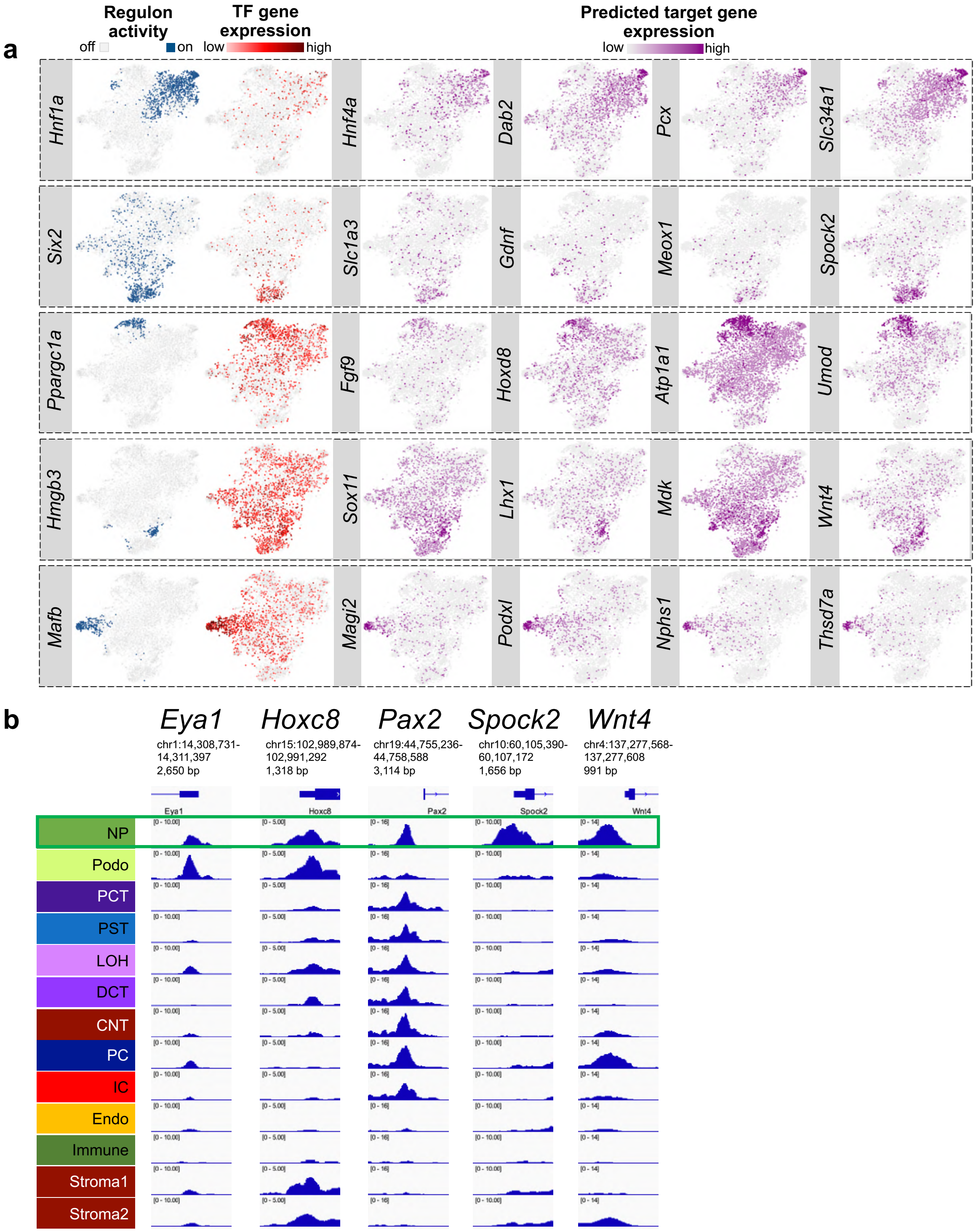

Supplementary Figure 15. Integrative analysis of transcription factors and target genes.
(a) UMAP depiction of regulon activity ("on-blue", "off-grey") and RNA expression (red scale) of exemplary regulons of proximal tubule (*Hnf1a*), nephron progenitors (*Six2*), loop of Henle (*Ppargc1a*), proliferating cells (*Hmgb3*) and podocytes (*Mafb*), respectively. Exemplary target gene expression for the respective TF is shown in purple scale. (b) Cell type-specific chromatin accessibility around transcription start sites of SCENIC-predicted target genes of the *Uncx* regulon (corresponding to **Figure 2d**). Genomic location and distance measures are given at the top.

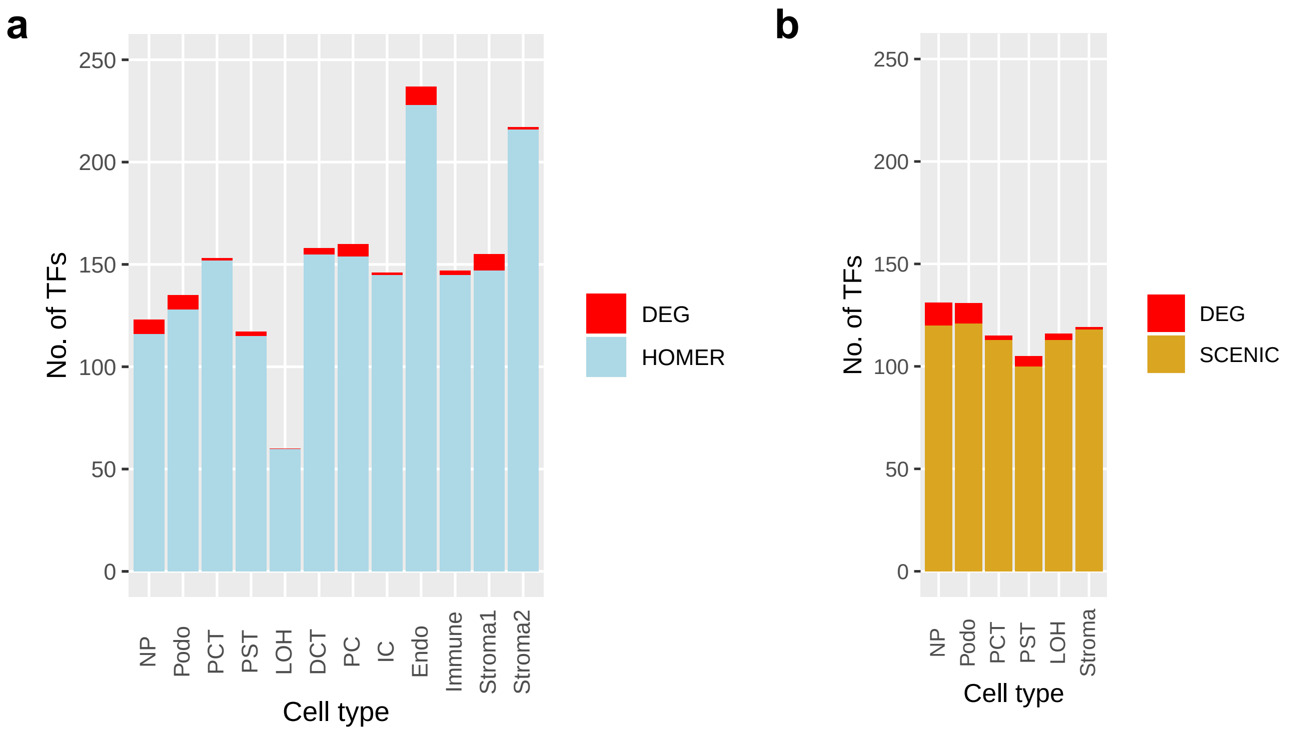

Supplementary Figure 16. Comparison of computationally inferred cell type-specific TFs.
Bar graphs depicting the absolute number of cell type-specific TFs reported by HOMER (a) and SCENIC (b) cis-regulatory analyses, respectively, as well as the number of TFs among DEGs from RNA expression data alone.

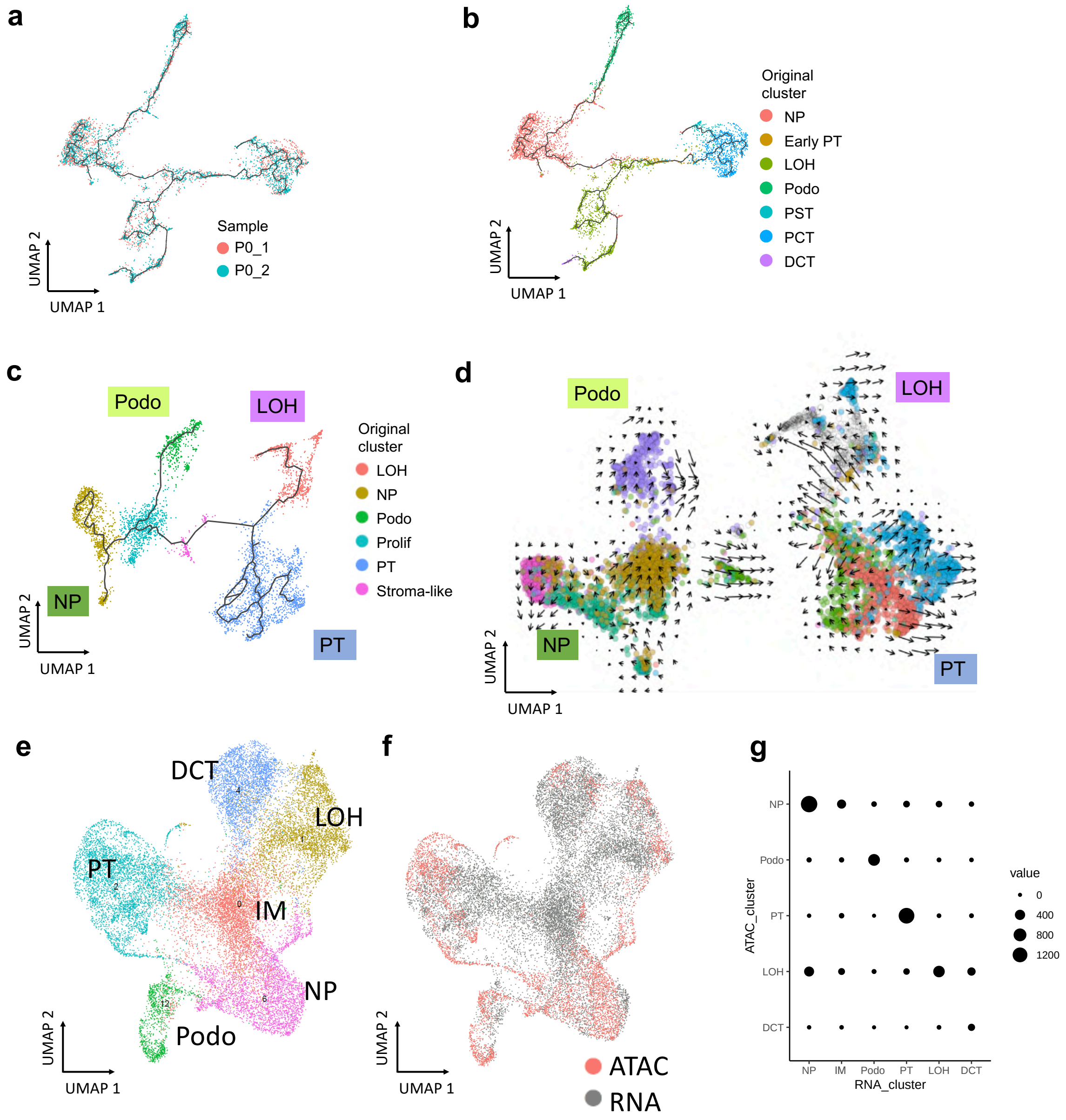

Supplementary Figure 17. snATAC-seq and scRNA-seq cell differentiation trajectories.
(a) UMAP representation of snATAC-seq trajectory lineages of podocytes, proximal tubule and loop of Henle cells from nephron progenitors colored by 2 P0 batches. (b) UMAP representation of snATAC-seq trajectory lineages of podocytes, proximal tubule and loop of Henle cells from nephron progenitors colored by original cell type assignment as in **Figure 1b**. (c) UMAP representation of scRNA-seq trajectory lineages of podocytes, proximal tubule and loop of Henle cells from nephron progenitors colored by original cell type assignment as in **Figure 1b**. (d) UMAP representation of RNA velocity of scRNA-seq trajectory inferred by VelocytoR, colored by original cell type assignment. Each dot is one cell and each arrow represents the time derivative of the gene expression state. (e) UMAP representation of snATAC-scRNA integration results colored by cell type assignment. (f) UMAP representation of snATAC-scRNA integration results colored by technologies (snATAC=red, scRNA=grey). Podo: podocytes, PT: proximal tubule, LOH: loop of Henle, DCT: distal convoluted tubule, NP: nephron progenitors, IM: intermediate stage cells. (g) Dot plot showing snATAC-scRNA integration cell type assignment confusion matrix. Each column represents the original cell type assignment of snATAC-seq data, and each row represents the predicted cell type assignment by the integration analysis scRNA-seq data. Each dot represents the number of cells that were matched in the integrated data.

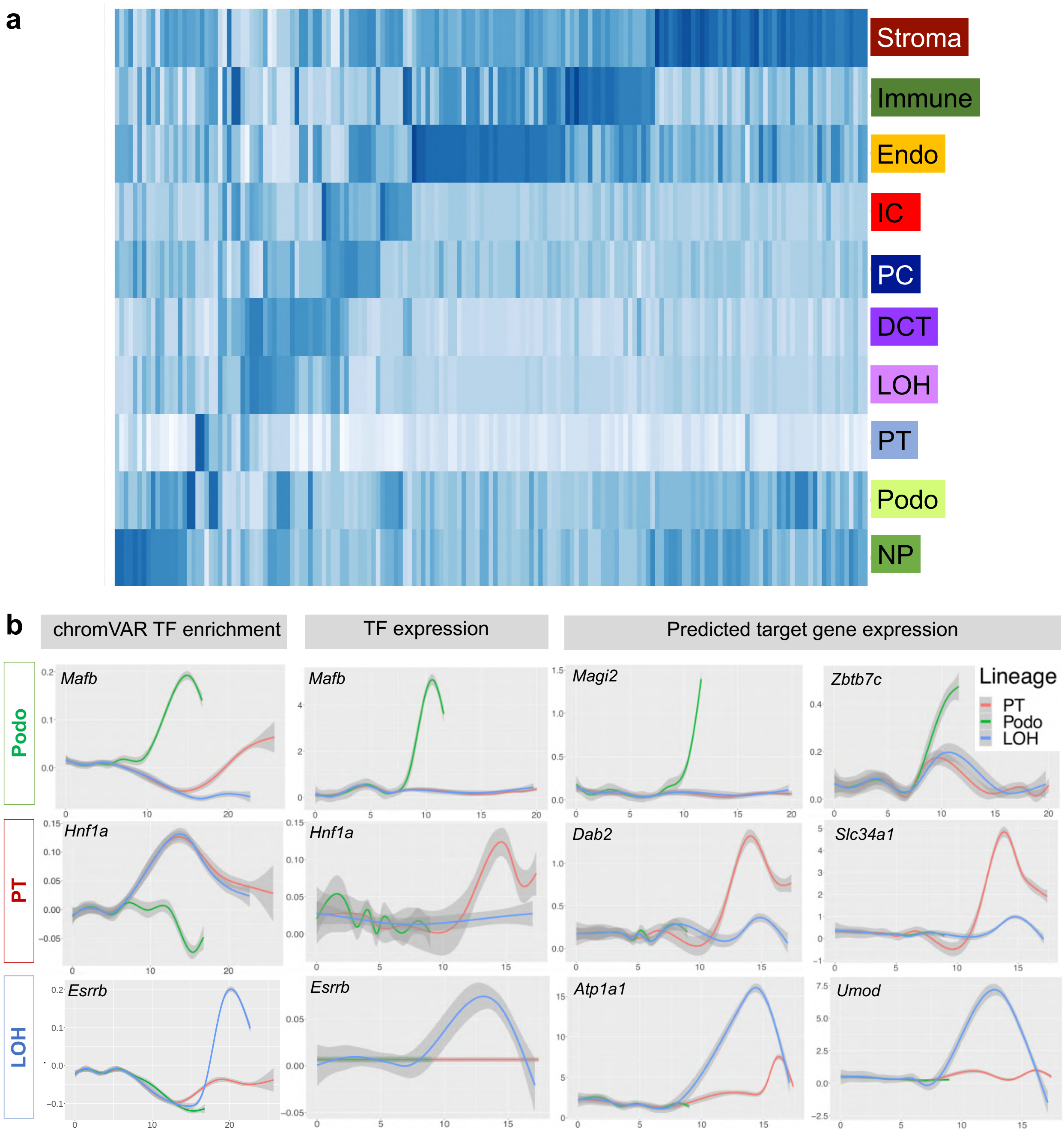

Supplementary Figure 18. Cell type-specific dynamics of motif enrichment and transcription factor expression.

(a) Heatmap of chromVAR enrichment results. The original data matrix is given in **Supplementary Data 9**. (b) Pseudotime-dependent chromatin accessibility and gene expression changes along the proximal tubule (red), podocytes (green) and loop of Henle (blue) cell lineages. The first column represents the dynamics of chromVAR TF enrichment score, the second column represents the dynamics of TF gene expression values, and the third and fourth column represent the dynamics of SCENIC-reported target gene expression values. Error bands denote 95% confidence intervals of local polynomial regression fitting.

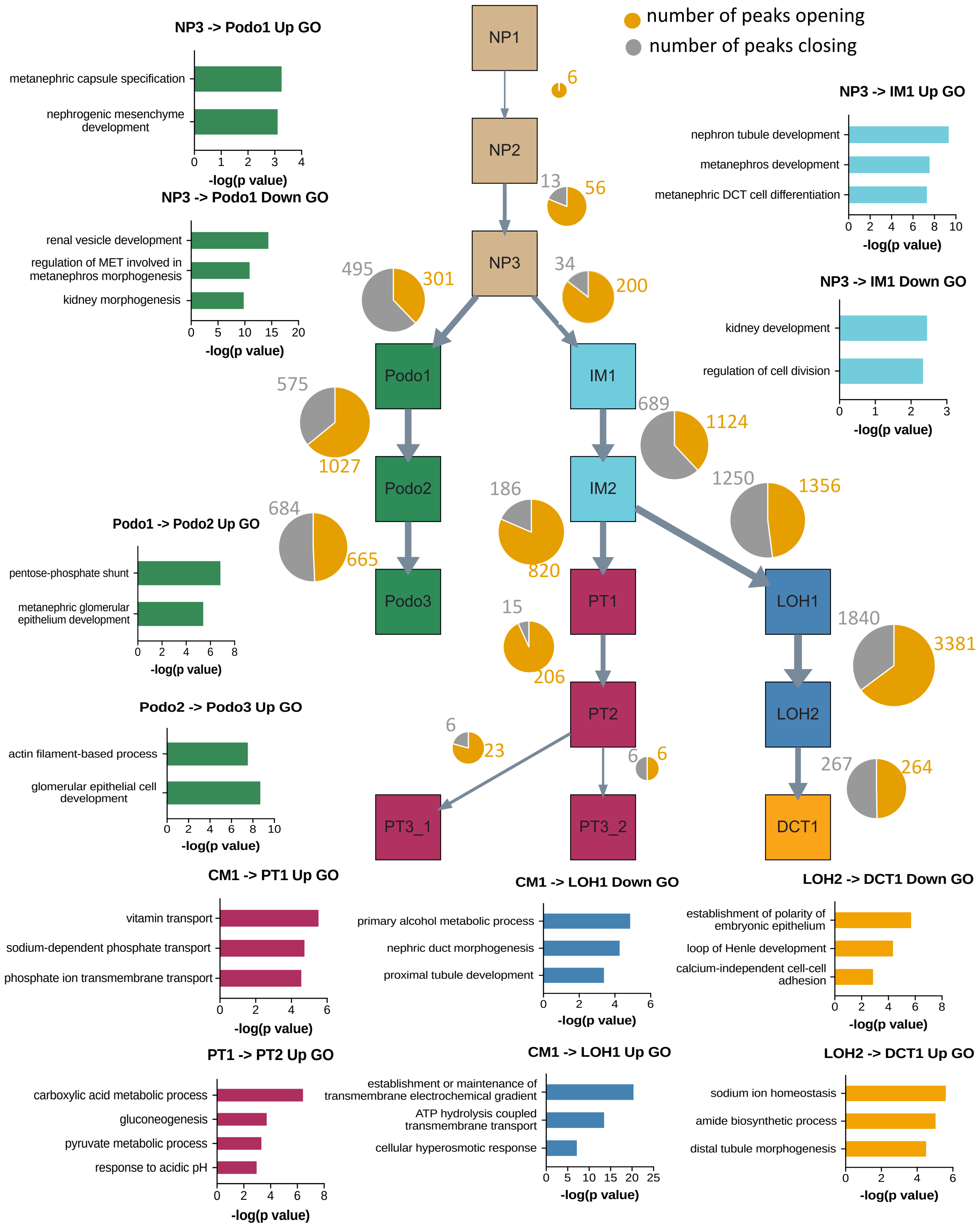

Supplementary Figure 19. Chromatin dynamics of nephron progenitor differentiation.
Di-graph representing cell type and lineage divergence, as derived from Cicero trajectory inference. Nephron progenitors (NP), podocytes (Podo), intermediate stage (IM), proximal tubule (PT), loop of Henle (LOH) and distal convoluted tubule (DCT) are connected with their developmental precursor stages and ordered by ascending numbering. Pie charts represent differentially assessible peaks (DAPs) between two stages, where the size of pie charts is proportional to the number of DAPs, orange color represents the number of open peaks, grey color the number of closed peaks. Bar graphs depict gene ontology (GO) term analysis of genes nearby DAPs derived from GREAT analysis (full list in **Supplementary Data 13**).

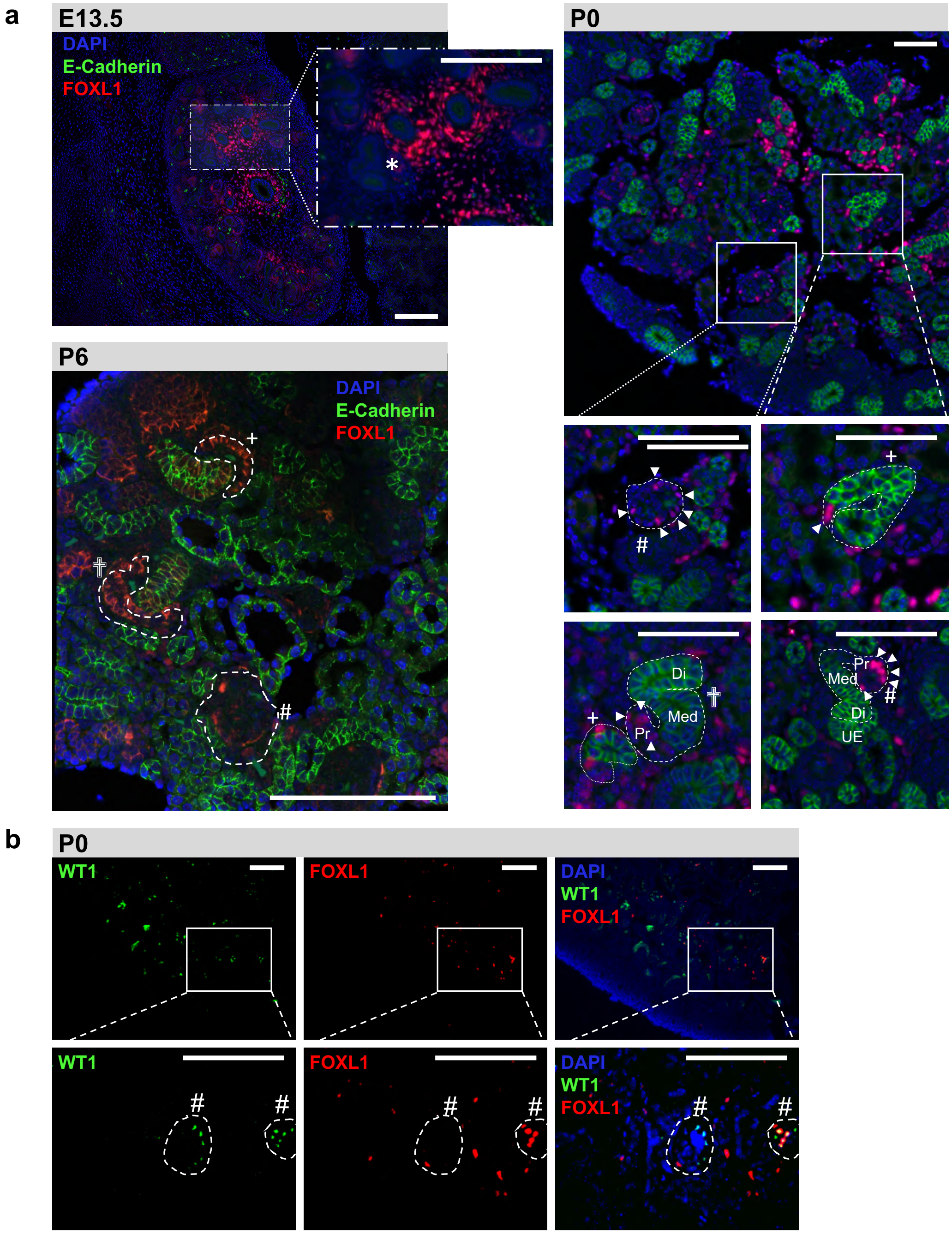

Supplementary Figure 20. FOXL1 protein expression during kidney development.

(a) Immunofluorescence staining of fetal mouse kidney. Upper left panel and insert denote E13.5 stage, right panel denotes P0, lower left panel denotes P6 mouse. Blue staining represents nuclei (DAPI), green staining represents tubular epithelium (E-Cadherin) and red staining represents progenitor cells (FOXL1) along a developmental trajectory from early progenitor stage (asterisk) over comma-shaped (+) and S-shaped bodies (cross) towards podocytes within glomeruli (#). Arrowheads denote FOXL1 positive cells in P0. Pr, proximal part of S-shaped body; Med, medial part of S-shaped body; Di, distal part of S-shaped body; UE, ureteric epithelium. Images are representative of 3 independent experiments; scale bar = 50 µm. (b) Immunofluorescence staining of P0 mouse kidney confirming a nuclear pattern of FOXL1 expression (red) co-localizing with WT1 expression (green) as a surrogate marker for podocytes. Merged images with nuclear staining represented by DAPI are on the right. Dashed lines in zoom-in versions marked # represent glomeruli. Images are representative of 3 independent experiments; scale bar = 50 µm.

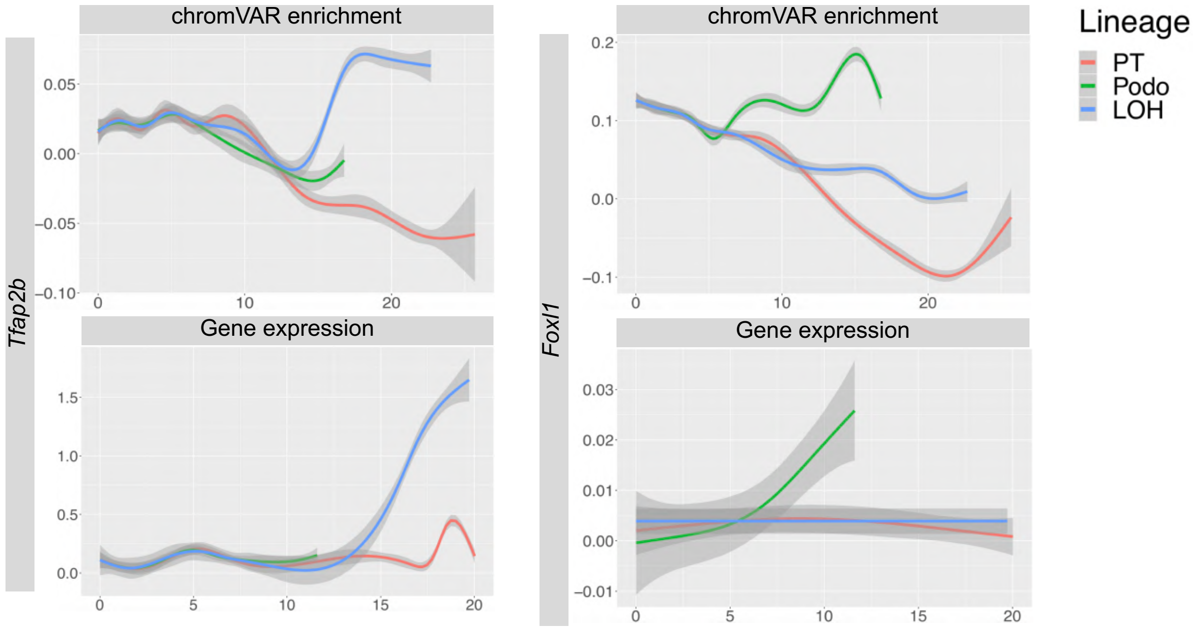

Supplementary Figure 21. *Foxl1* and *Tfap2b* expression and chromVAR enrichment along the developmental lineages.

Pseudotime-dependent chromatin accessibility and gene expression changes along the proximal tubule (red), podocytes (green) and loop of Henle (LOH, blue) cell lineages for important bifurcation TFs in the podocyte (*Foxl1*) and distal tubule (*Tfap2b*) lineage. Error bands denote 95% confidence intervals of local polynomial regression fitting.

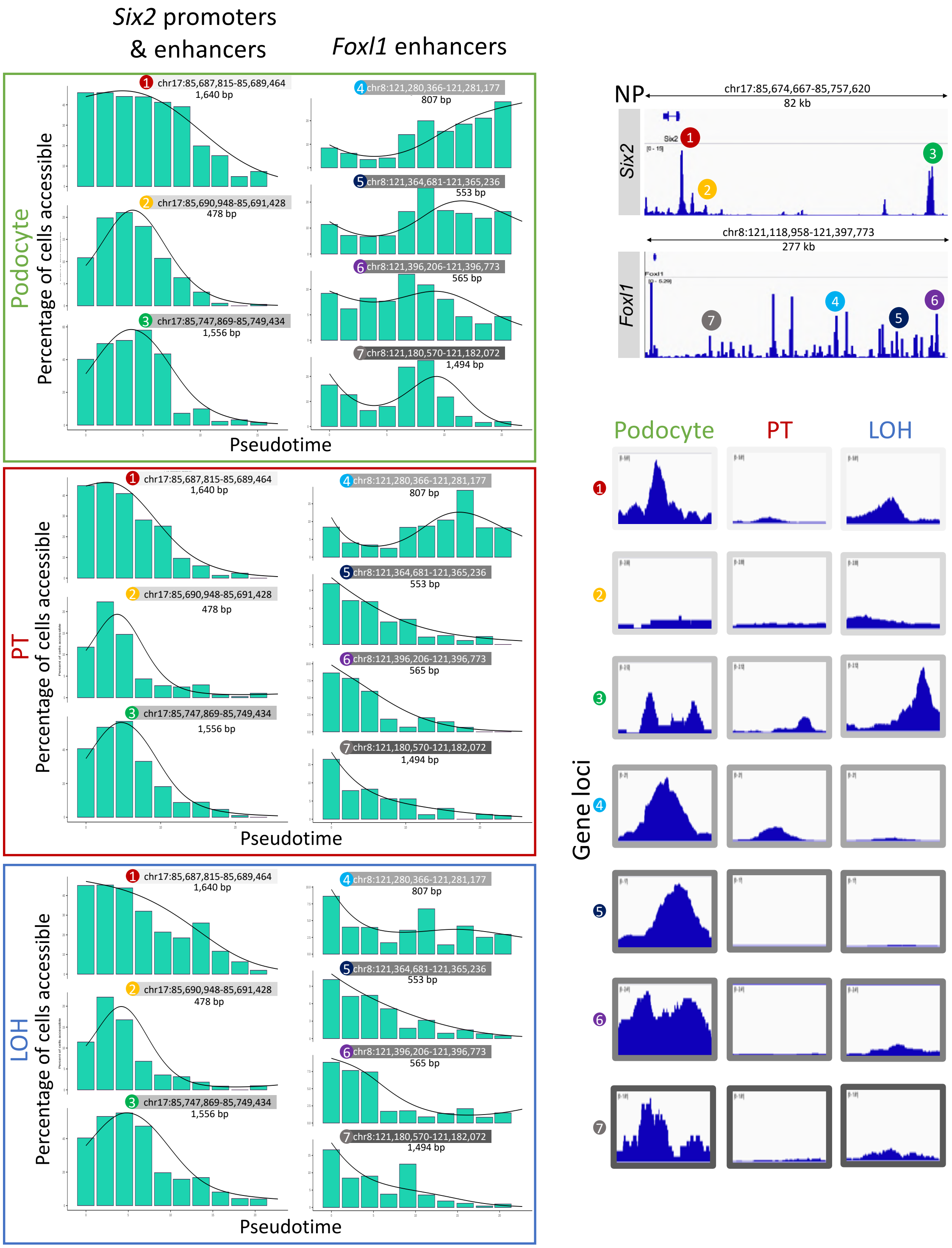

Supplementary Figure 22. Chromatin accessibility of *Six2* promoters and enhancers as well as putative *Foxl1* enhancers.

Bar graphs denote the percentage of cells with accessible chromatin of several *Six2* promoters and enhancers (gene loci numbered 1-3) as well as putative *Foxl1* enhancers (gene loci numbered 4-7) along pseudotime. Exact gene loci of enhancers and promoters are given above each respective graph. Changes along pseudotime are depicted for 3 lineages from nephron progenitors (NP) to podocytes, proximal tubule (PT) and loop of Henle (LOH) cells, respectively. The right upper subpanel depicts the genome browser overview of chromatin accessibility for the NP and therefore corresponds to the first bar in graphs on the left. The right lower subpanel depicts zoom-in versions of the 7 loci for all 3 lineages.

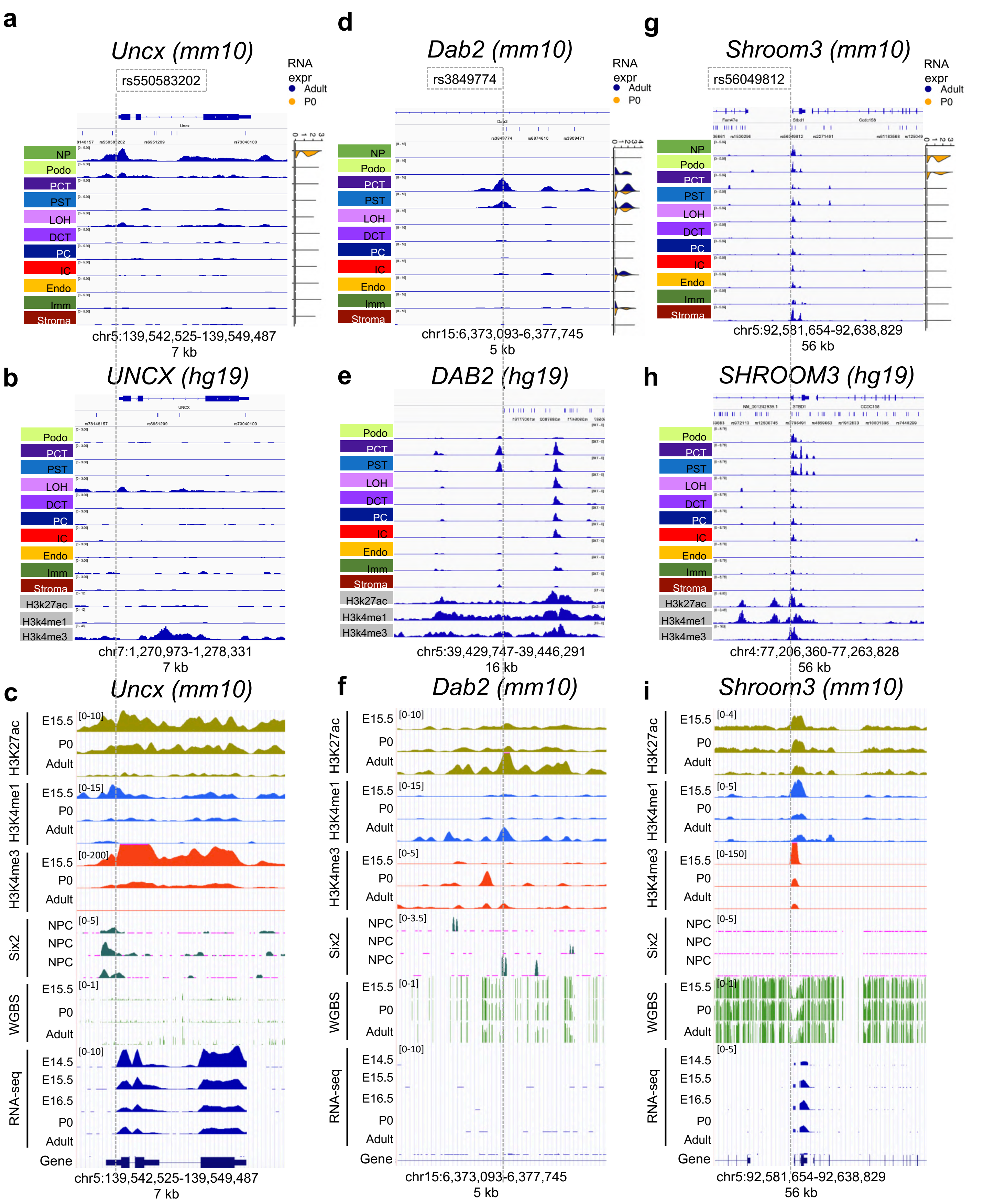
a
Uncx (mm10)
rs550583202
RNA expr
Adult
P0
NP
Podo
PCT
PST
LOH
DCT
PC
IC
Endo
Imm
Stroma
chr5:139,542,525-139,549,487
7 kb
d
Dab2 (mm10)
rs3849774
chr15:6,373,093-6,377,745
5 kb
g
Shroom3 (mm10)
rs56049812
chr5:92,581,654-92,638,829
56 kb
b
UNCX (hg19)
H3k27ac
H3k4me1
H3k4me3
chr7:1,270,973-1,278,331
7 kb
e
DAB2 (hg19)
chr5:39,429,747-39,446,291
16 kb
h
SHROOM3 (hg19)
chr4:77,206,360-77,263,828
56 kb
c
Uncx (mm10)
H3K27ac
H3K4me1
H3K4me3
Six2
WGBS
RNA-seq
E15.5
P0
Adult
NPC
E14.5
E16.5
Gene
f
Dab2 (mm10)
i
Shroom3 (mm10)

Supplementary Figure 23. Single cell level chromatin accessibility highlighted human kidney GWAS target genes and cell types.

Open chromatin and co-accessibility view at alternative, more zoomed-in scales to those shown in **Figure 6**.
(a, d, g) Genome browser view of *Shroom3*, *Dab2* and *Uncx* loci; from top to bottom: mouse orthologue of eGFR GWAS significant SNPs (after lift-over) mouse kidney single nuclei chromatin accessibility for nephron progenitors (NP), podocytes (Podo), proximal convoluted and straight tubules (PCT and PST), loop of Henle (LOH), distal convoluted tubule (DCT), collecting duct intercalated cells (IC), collecting duct principal cell types (PC), endothelial cells (Endo), immune cells (Immune) and stromal cells (Stroma). Data range in all tracks is set to the same scale. Examples of cell type-specific accessible chromatin overlapped with significant SNPs are highlighted with dashed lines. Right subpanel shows violin plots of cell type-specific gene expression in P0 (orange) and adult (blue) kidneys in the scRNA-seq dataset. (b, e, h) Corresponding genome browser views in adult human (hg19) kidney snATAC-seq data; from top to bottom: eGFR GWAS significant SNPs, adult human kidney single nuclei chromatin accessible landscape, whole kidney H3K27ac, H3K4me1 and H3K4me3 ChIP-seq tracks. The genomic location in human was matched to the mouse orthologue. Note that for *DAB2* (f), the image was mirrored to facilitate comparison along genomic read direction. (c, f, i) Whole mouse kidney epigenomics tracks from E14.5, E15.5, E16.5, P0 and adult mice. From top to bottom: H3K27ac, H3K4me1, H3K4me3, and Six2 ChIP-seq; whole genome bisulfate sequencing (WGBS); and bulk RNA-seq. The bottom Refseq visualization corresponds to the Refseq track at the top in (a, d, g), respectively. Six2 binding signal in nephron progenitor cells.
