# Supplementary material for: Single cell regulatory landscape of the mouse kidney highlights cellular differentiation programs and disease targets
Source: Nat Commun. 2021 Apr 15;12:2277. doi: 10.1038/s41467-021-22266-1 (PMC8050063; doi:10.1038/s41467-021-22266-1)
Supplement: Supplementary file 3 — Description of Additional Supplementary Files [file 41467_2021_22266_MOESM3_ESM.pdf]

Description of Additional Supplementary Files

File Name: Supplementary Data 1
Description: Quality control and metadata information of scRNA-seq data.

File Name: Supplementary Data 2
Description: Cell type marker genes derived from scRNA-seq analysis & marker genes used for annotation.

File Name: Supplementary Data 3
Description: DEG in scRNA-seq data after ambient RNA cleaning.

File Name: Supplementary Data 4
Description: DEG in scRNA-seq data of stroma subclusters.

File Name: Supplementary Data 5
Description: Cell type-specific open chromatin derived from snATAC-seq analysis.

File Name: Supplementary Data 6
Description: Cell type-specific motif enrichment.

File Name: Supplementary Data 7
Description: Regulons and respective target genes inferred by SCENIC.

File Name: Supplementary Data 8
Description: Scaled and binarized regulon activities in each cell type inferred by SCENIC.

File Name: Supplementary Data 9
Description: ChromVAR cell-TF enrichment score matrix.

File Name: Supplementary Data 10
Description: DEG along pseudotime in distinct lineages in scRNA-seq data.

File Name: Supplementary Data 11
Description: Differentially accessible peaks along pseudotime in distinct lineages in snATAC-seq data.

File Name: Supplementary Data 12
Description: Nearest genes of differentially accessible peaks along pseudotime in distinct lineages in snATAC-seq data.

File Name: Supplementary Data 13
Description: GO enrichment of differentially accessible peaks along pseudotime in distinct lineages inferred by GREAT analysis.

File Name: Supplementary Data 14
Description: Nearest genes of differentially accessible peaks at bifurcation events along pseudotime in distinct lineages inferred by GREAT analysis.

File Name: Supplementary Data 15
Description: Proportion of cells in each cell type with accessible chromatin overlapped with kidney disease-associated SNPs.

File Name: Supplementary Data 16
Description: Material Table
