# Supplementary material for: Single cell regulatory landscape of the mouse kidney highlights cellular differentiation programs and disease targets
Source: Nat Commun. 2021 Apr 15;12:2277. doi: 10.1038/s41467-021-22266-1 (PMC8050063; doi:10.1038/s41467-021-22266-1)
Supplement: Supplementary file 20 — Reporting Summary [file 41467_2021_22266_MOESM20_ESM.pdf]

nature research

Corresponding author(s): Katalin Susztak

Last updated by author(s): Jan 18, 2020

Reporting Summary

Nature Research wishes to improve the reproducibility of the work that we publish. This form provides structure for consistency and transparency in reporting. For further information on Nature Research policies, see our Editorial Policies and the Editorial Policy Checklist.

Statistics

For all statistical analyses, confirm that the following items are present in the figure legend, table legend, main text, or Methods section.

n/a	Confirmed	
☐	☒	The exact sample size (*n*) for each experimental group/condition, given as a discrete number and unit of measurement
☐	☒	A statement on whether measurements were taken from distinct samples or whether the same sample was measured repeatedly
☐	☒	The statistical test(s) used AND whether they are one- or two-sided<br>*Only common tests should be described solely by name; describe more complex techniques in the Methods section.*
☒	☐	A description of all covariates tested
☐	☒	A description of any assumptions or corrections, such as tests of normality and adjustment for multiple comparisons
☐	☒	A full description of the statistical parameters including central tendency (e.g. means) or other basic estimates (e.g. regression coefficient) AND variation (e.g. standard deviation) or associated estimates of uncertainty (e.g. confidence intervals)
☐	☒	For null hypothesis testing, the test statistic (e.g. *F*, *t*, *r*) with confidence intervals, effect sizes, degrees of freedom and *P* value noted<br>*Give P values as exact values whenever suitable.*
☒	☐	For Bayesian analysis, information on the choice of priors and Markov chain Monte Carlo settings
☒	☐	For hierarchical and complex designs, identification of the appropriate level for tests and full reporting of outcomes
☐	☒	Estimates of effect sizes (e.g. Cohen's *d*, Pearson's *r*), indicating how they were calculated

Our web collection on statistics for biologists contains articles on many of the points above.

Software and code

Policy information about availability of computer code

Data collection	Cell Ranger ATAC v. 1.1.0 – Barcode Identification, Alignment, Filter, Deduplication<br>Cell Ranger v. 3.1.0 – Barcode Identification, Alignment, Filter, Deduplication
Data analysis	bedtools v. 2.29.2<br>CellPhoneDB v. 2.1.2<br>ChromVAR v. 3.1.0<br>Cicero v. 1.5.5<br>deeptools v. 2.0<br>ENCODE ATAC-seq pipeline v. 1.9.1<br>GenomicRanges v. 1.40.0<br>GREAT v. 4.0.4<br>Harmony v. 1.0<br>HOMER v. 4.10.4<br>IGV v. 2.8.9<br>MACS2 v. 2.2.6<br>Monocle3 v. 0.1.3<br>R v. 3.5.1<br>SCENIC v. 1.1.2.2<br>Seurat v. 3.0<br>SeuratWrappers v. 1.0<br>snapATAC v. 1.0<br>SoupX v. 1.4.5<br>UCSC bedgraphtobigwig v. 2.8<br>UCSC liftOver v.1.14.0

VelocytoR v. 0.17
VisCello v. 1.0.0
Custom codes (https://github.com/Zhen-Miao/dev-kidney-snATAC)

For manuscripts utilizing custom algorithms or software that are central to the research but not yet described in published literature, software must be made available to editors and reviewers. We strongly encourage code deposition in a community repository (e.g. GitHub). See the Nature Research guidelines for submitting code & software for further information.

Data

Policy information about availability of data

All manuscripts must include a data availability statement. This statement should provide the following information, where applicable:

- Accession codes, unique identifiers, or web links for publicly available datasets
- A list of figures that have associated raw data
- A description of any restrictions on data availability

Raw data, processed data, and metadata from mouse samples are available at GEO under accession GSE157079. The processed data and metadata can also be viewed, analyzed, and downloaded at susztaklab.com/developing_adult_kidney/snATAC/, susztaklab.com/developing_adult_kidney/scRNA/, and susztaklab.com/developing_adult_kidney/igv/. The raw data, processed data, and metadata from human samples are available at https://www.diabetesepigenome.org.
In this study, we downloaded public data from the following database with accession numbers: GUDMAP (RID:Q-Y4CY); ENCODE (ENCFF338WZP, ENCFF872MVE, ENCFF455HPY, ENCFF049LRQ, ENCFF179NTO, ENCFF071PID, ENCFF746MFH, ENCFF563LOO, ENCFF184AYF, ENCFF107NQP, ENCFF465THI, ENCFF769XWI, ENCFF591DAX); GEO (GSM1051156, GSM3716711, GSM3716714, GSM1586397)

Further information and requests for resources and reagents should be directed to and will be fulfilled by the lead contact: Katalin Susztak. email: ksusztak@pennmedicine.upenn.edu.

Field-specific reporting

Please select the one below that is the best fit for your research. If you are not sure, read the appropriate sections before making your selection.

☒ Life sciences ☐ Behavioural & social sciences ☐ Ecological, evolutionary & environmental sciences

For a reference copy of the document with all sections, see nature.com/documents/nr-reporting-summary-flat.pdf

Life sciences study design

All studies must disclose on these points even when the disclosure is negative.

Sample size	No statistical methods were used to pre-determine sample size. Post hoc analysis showing high reproducibility and the agreement of cell type clusters. We compared the number of cells profiled in other published dataset to choose the sample size for this study.
Data exclusions	No data were excluded in this study.
Replication	All results presented in manuscript were reliably reproduced. Wet lab experiments are representative of 3 independent experiments.
Randomization	No randomization was used in human sample collection, wild type mice were randomly chosen for experiments.
Blinding	Blinding was not relevant with this type of analysis, we collected samples that were available to us. Investigators were blinded to allocation during experiments and outcome assessments.

Reporting for specific materials, systems and methods

We require information from authors about some types of materials, experimental systems and methods used in many studies. Here, indicate whether each material, system or method listed is relevant to your study. If you are not sure if a list item applies to your research, read the appropriate section before selecting a response.

Materials & experimental systems

n/a	Involved in the study	
☐	☒	Antibodies
☒	☐	Eukaryotic cell lines
☒	☐	Palaeontology and archaeology
☐	☒	Animals and other organisms
☐	☒	Human research participants
☒	☐	Clinical data
☒	☐	Dual use research of concern

Methods

n/a	Involved in the study	
☒	☐	ChIP-seq
☒	☐	Flow cytometry
☒	☐	MRI-based neuroimaging

Antibodies

Antibodies used	Guinea pig anti-FOXL1 (own production)<br>Mouse anti-E-Cadherin, clone 36 (BD Transduction Lab, 610182)<br>Goat anti-WT1, clone F-6 (Santa Cruz, sc-7385)<br>Cy2-conjugated donkey secondary antibody (Jackson ImmunoResearch Laboratories, 715-225-150)<br>Cy3-conjugated donkey secondary antibody (Jackson ImmunoResearch Laboratories, 706-165-150)<br>Cy5-conjugated donkey secondary antibody (Jackson ImmunoResearch Laboratories, 705-175-150)<br>AlexaFluor 488-conjugated donkey secondary antibody (LifeSciences, A11029)
Validation	All antibodies were validated by the manufacturer or in prior publications: Aoki et al. (doi: 10.1016/j.jcmgh.2015.12.004), https://www.bdbiosciences.com, https://datasheets.scbt.com/sc-7385.pdf, https://www.jacksonimmuno.com/catalog/products/715-225-150, https://www.jacksonimmuno.com/catalog/products/715-165-150, https://www.jacksonimmuno.com/catalog/products/715-175-150, https://www.thermofisher.com/antibody/product/Goat-anti-Mouse-IgG-H-L-Highly-Cross-Adsorbed-Secondary-Antibody-Polyclonal/A-11029

Animals and other organisms

Policy information about studies involving animals; ARRIVE guidelines recommended for reporting animal research

Laboratory animals	Wild type mice (C57BL/6) were used in this study, the age of the mice are 1 day, 3-week, and 8-week old. For the snATAC-seq study, all samples were collected from female mice.
Wild animals	This study did not involve wild animals.
Field-collected samples	This study did not involve samples collected from the field.
Ethics oversight	The animal experiments were reviewed and approved by the Institutional Animal Care and Use Committee (IACUC) of the University of Pennsylvania in accordance with the guidelines of the National Institutes of Health.

Note that full information on the approval of the study protocol must also be provided in the manuscript.

Human research participants

Policy information about studies involving human research participants

Population characteristics	We conducted single-nuclei ATAC-seq experiments with healthy human kidneys from surgical nephrectomies from 6 patients. From patient 1 to 6, the ages are 52, 70, 85, 85, 74,65; the genders are Male,Male,Female,Female,Male,Female; and the Races are European, European, European, European, African American, European.
Recruitment	Kidney samples were obtained from surgical nephrectomies approved by the University of Pennsylvania Institutional Review Board. Nephrectomies were de-identified, and the corresponding clinical information was collected through an honest broker via CHTN.
Ethics oversight	Institutional Review Boards at the University of Pennsylvania reviewed this study. This project utilized de-identified kidney biospecimens collected via CHTN (Cooperative Human Tissue Network) and therefore was considered “exempt” by the local IRB. The work was completed in compliance with all relevant ethical regulations.

Note that full information on the approval of the study protocol must also be provided in the manuscript.
